# Research progress on nationally protected plants in the three most north-eastern provinces of China

**DOI:** 10.3897/BDJ.13.e144780

**Published:** 2025-03-20

**Authors:** Yuzhu Sui, Hongfeng Wang

**Affiliations:** 1 Northeast Forestry University, Harbin, China Northeast Forestry University Harbin China; 2 Northeast Asia Biodiversity Research Center, Harbin, China Northeast Asia Biodiversity Research Center Harbin China

**Keywords:** China, nationally protected plants, distribution characteristics, threatened status, research status

## Abstract

**Background:**

The three most north-eastern provinces of China (Heilongjiang, Jilin, and Liaoning) are rich in natural resources and have favourable geographical conditions, making them home to a large number of wild plant species. This paper summarises the distribution characteristics, endangerment degree and research status of nationally protected plants in these provinces based on online databases, literature review and field surveys.

**New information:**

The results show that the north-eastern provinces have 31 families, 38 genera and 51 species of nationally protected plants. The endangered status includes both the endangered assessments made by China and those made globally. China has assessed 38 plant species as endangered, while 19 plant species are assessed as endangered globally. Currently, most of the protected plants have been studied, with in-situ and ex-situ conservation being the primary protective measures. In addition, this study also identified seven species of plants that are basically not covered by research and lack sufficient studies in the current literature and urgently need further in-depth investigation and long-term monitoring in order to improve the relevant conservation measures. This study provides a scientific basis for the conservation vacancies of plants under national key protection in the three north-eastern provinces, as well as a reference for formulating effective conservation policies and promoting further research.

## Introduction

China is one of the most biodiverse countries in the world, with unique ecosystems and rich species resources (State Council Information Office of the People's Republic of China 2021). Amongst them, the three most north-eastern provinces (Heilongjiang, Jilin and Liaoning) are rich in natural resources, not only possessing vast forest, wetland and grassland ecosystems, but also a large number of rare and endangered species, especially wild plants under national key protection (Fan et al. 2018). However, in recent years, the biodiversity of the three most north-eastern provinces is facing serious threats due to the intensification of human activities and changes in the natural environment (He 2020). Factors such as ecosystem degradation, habitat loss, climate change and invasion of exotic species have led to the deterioration of the living environment of many nationally key protected plants and a sharp decline in their populations (Ministry of Ecology an Environment of the People's Republic of China 2024).

According to the priorities in the Kunming-Montreal Global Biodiversity Framework (2022), which proposes key targets to conserve ecosystems, to reduce habitat loss and to prevent species extinction (United Nations Environment Programme (UNEP) and Convention on Biological Diversity (CBD) 2023), the three most north-eastern provinces are working to implement these targets, especially in flora conservation and restoration (Cao et al. 2022). Strengthening in-situ conservation and restoration of endangered plants, responding to climate change and enhancing habitat conservation (Yang et al. 2019) are all key initiatives in the three most north-eastern provinces in response to the Global Biodiversity Conservation Strategy (Ministry of Ecology an Environment of the People's Republic of China 2024). These initiatives not only help to improve the local biodiversity conservation environment, but also help to provide a model and experience for global biodiversity conservation.

In 1999, the State Forestry and Grassland Administration and the Ministry of Agriculture and Rural Development jointly released the first edition of the List of Wild Plants under State Key Protection (Ministry of National Forestry and Ministry of State for Agriculture 1999) and the situation of wild plant protection in China has changed greatly, with some endangered wild plants being effectively protected (State Forestry and Grassland Administration 2021). However, some wild plants have become more endangered due to habitat destruction and over-utilisation (State Forestry and Grassland Administration 2021). Therefore, with the approval of the State Council, the State Forestry and Grassland Administration and the Ministry of Agriculture and Rural Development of the People's Republic of China, the latest version of the List of Wild Plants under State Key Protection was released in 2021 to address biodiversity conservation in the current situation (National Forestry and Grassland Administration 2021).

Flora research in the three most north-eastern provinces began in the mid-19^th^ and 20^th^ centuries, with Russian and Japanese scholars becoming the main research force (Cao et al. 2022). Early studies focused on basic investigations of plant classification and composition of the flora, such as the compilation of the Northeastern Plant Search List (Fu 1995). With the development of floristics, the focus of research gradually shifted to the origin and evolution of flora and its relationship with the environment, for example, the detailed analyses of Cao Wei et al. (Cao and Li 2008) of the vertical distribution pattern of the flora in the Changbai Mountains revealed the process of flora's adaptation and evolution in response to environmental changes. In recent years, flora research in the three most north-eastern provinces has made remarkable progress in terms of methodology and technology and the application of new technologies, such as molecular biology, remote sensing technology and geographic information systems (GIS), has provided strong support for in-depth studies of flora (Wang 2023).

There are many rare and endangered plants under national key protection in the three most north-eastern provinces, especially *Taxuscuspidata*, *Thujakoraiensis* and Orchidaceae. *Taxuscuspidata* is a national-level protected plant and a very small population of wild plants, mainly distributed in the north-eastern region of China (Institute of Botany, Chinese Academy of Sciences 2013), which has become an endangered species because of its narrow distribution area, serious habitat fragmentation and decreasing natural distribution (Liu 2024). *Taxuscuspidata* has become an endangered species. At present, the protection of *Taxuscuspidata* counters is mainly based on in-situ protection, supplemented by field return and relocation protection (Zhu et al. 2023). *Thujakoraiensis*, is only distributed in China in the Changbai Mountain area of Jilin Province, with low seed viability, coupled with over-exploitation, the geographical distribution of natural populations has greatly reduced and the natural regeneration capacity is very weak (Jin et al. 2019) and a nature reserve for *Thujakoraiensis* has been set up in the Changbai Mountain area (Yin et al. 2019). Orchidaceae is the family with the largest number of threatened species amongst angiosperms and also the family with the largest number of extinct species (Qin et al. 2017) and, due to the destruction of habitats, the existing Orchidaceae resources in northeast China have gradually decreased and, at present, Orchidaceae plants in northeast China are mainly zoned for protection in the habitats where they are located (Xu 2024).

In this paper, 51 species of plants under state key protection in the three most north-eastern provinces of China were studied and the list of plants under state key protection in the three most north-eastern provinces was compiled, along with their distribution, threat level, threat factors and research status through literature, online database and field survey. This study aims to account for the distribution pattern of plants under state key protection in the three most north-eastern provinces, assess the endangered status of plants under state key protection in the three most north-eastern provinces and point out the research vacancies of plants under state key protection in the three most north-eastern provinces. The results of this study provide a scientific basis for the conservation vacancies of national key protected plants in the three most north-eastern provinces and, at the same time, the study provides a reference for the formulation of an effective conservation policy, which helps to promote further research on national key protected plants in the three most north-eastern provinces.

## Materials and methods

### Data sources

The main sources of data on the list and distribution of nationally protected plants in the three most north-eastern provinces include the "National Key Protected Wild Plants List (2021)" (National Forestry and Grassland Administration 2021), "The List of Wild Vascular Plants in Heilongjiang Province" (Wang et al. 2022), "The List of National Key Protected Wild Plants in Jilin Province (2021)" (Jilin Provincial Forestry and Grassland Administration 2021), "Catalogue of Higher Plant Diversity in Liaoning Province" (Zhang et al. 2022), the "Jilin Forestry and Grassland Bureau’s wild plant column" (Jilin Provincial Forestry and Grassland Administration 2023) and relevant literature on 51 protected plants. The information derived from these references were compiled to create an overall list for these three provinces as a base for further investigations.

The protection levels of national protected plants in the three most north-eastern provinces are based on Article 2 of the "Wild Plant Protection Regulations of the People's Republic of China", (National Forestry and Grassland Administration 2017) which states: "The wild plants protected by these regulations refer to rare plants that grow naturally in their native habitat, as well as endangered and rare plants that grow naturally in their native habitat and have significant economic, scientific research or cultural value".

Level I protected plants are those that are critically endangered, with extremely limited populations, narrow distribution and strict environmental requirements for survival. The protection of these plants is crucial, as failure to protect them could lead to their extinction. Typically, Level I protected plants are characterised by the following features:

1. Extremely small populations;
2. Very narrow distribution range;
3. Extremely high ecological, economic or cultural value;
4. Facing significant human activity threats.

Level II protected plants are those that, while facing some threats, have relatively stable populations and distribution and are not yet at immediate risk of extinction. These plants still require protection to some degree, but the protection measures can be somewhat more lenient compared to Level I protected plants. Level II protected plants are characterised by the following features:

1. Relatively stable populations;
2. Broader distribution range;
3. High ecological, economic or cultural value;
4. Threats are less severe compared to Level I protected plants.

The global endangered status of the national protected plants in the three most north-eastern provinces is determined according to the IUCN Red List Categories and Criteria (IUCN Species Survival Commission 2000). The endangered status categories include: Extinct (EX), Extinct in the Wild (EW), Critically Endangered (CR), Endangered (EN), Vulnerable (VU), Near Threatened (NT), Least Concern (LC), Data Deficient (DD) and Not Evaluated (NE).

The endangered status of the national protected plants in the three most north-eastern provinces of China is determined according to the "China Red List of Biodiversity - Higher Plants Volume" assessment report (Ministry of Ecology and Environment of People's Republic of China and Chinese Academy of Sciences 2013). The species' endangered status categories include: Extinct (EX), Extinct in the Wild (EW), Region Extinct (RE), Critically Endangered (CR), Endangered (EN), Vulnerable (VU), Near Threatened (NT), Least Concern (LC) and Data Deficient (DD).

The current research status of national protected plants in the three most north-eastern provinces is based on the Chinese and Latin names of 51 species of nationally protected plants distributed in the region as keywords. Literature searches were conducted in CNKI (China National Knowledge Infrastructure) and Web of Science. The search criteria were based on the research areas of the three north-eastern provinces, the north-eastern region, Heilongjiang Province, Jilin Province and Liaoning Province. The retrieved literature was then screened accordingly. The number of relevant publications and the related research fields for each species were determined.

### Data processing

The main steps for processing data include:

1. Unify the Latin names in the following references: "List of National Key Protected Wild Plants (2021)" (National Forestry and Grassland Administration 2021), "Checklist of Wild Vascular Plants in Heilongjiang Province" (Wang et al. 2022), "List of National Key Protected Wild Plants in Jilin Province (2021)" (Jilin Provincial Forestry and Grassland Administration 2021) and "Catalog of Vascular Plant Diversity in Liaoning Province" (Zhang et al. 2022).

2. Match the plant lists of Heilongjiang Province and Liaoning Province with the "List of National Key Protected Wild Plants (2021)" (National Forestry and Grassland Administration 2021) and compile the "List of National Key Protected Wild Plants in Heilongjiang Province" (Wang et al. 2022) and the "List of National Key Protected Wild Plants in Liaoning Province" (Zhang et al. 2022).

3. Based on the Wild Plant Column of the Jilin Provincial Forestry and Grassland Bureau Jilin Provincial Forestry and Grassland Administration 2023 (Jilin Provincial Forestry and Grassland Administration 2023) and relevant literature on 51 protected plant species, the distribution of the 51 protected plant species was compiled.

## Checklists

### Checklist of national key protected wild plants in the three most north-eastern provinces of China

#### Actinidiaceae

7046ED4F-3FB2-5DE3-B284-0BBF7DEB971D

#### Actinidia arguta

(Siebold & Zucc.) Planch. ex Miq.

B5DD3A0C-F265-5BD0-AB14-E0E58E6A8283

##### Distribution

**Heilongjiang Province**: Acheng District, Harbin City, Shangzhi City (Wang et al. 2022).

**Jilin Province**: Linjiang City, Helong City, Antu County, Fusong County, Wangqing County, Hunchun City, Jiaohe City, Ji'an City (Xiao et al. 2010, Li et al. 2015, Liu 2019b, Peng 2019).

**Liaoning Province**: Xifeng City, Qingyuan City, Huanren County, Fengcheng City, Benxi City, Xiuyan County, Zhuanghe City, Wafangdian District, Jinzhou District, Suizhong County (Liu and Zhu 2010, Zou 2017, Chang et al. 2020).

##### Notes

National Protected Plants (Level II).

(Fig. 1)

#### Alismataceae

E288CFF2-B66E-5359-B3DA-2B79BAAA2981

#### Sagittaria natans

Pall.

C03BEEE0-45F6-51C3-B66C-DA36EE9D4954

##### Distribution

**Heilongjiang Province**: Acheng District, Daxing'anling Prefecture, Harbin City, Jixi City, Shangzhi City, Yichun City, Zhaodong City, Zhaozhou County, Beian City, Heihe City, Huma County, Hulun City, Jiayin County, Mohe City (Wang et al. 2022).

**Jilin Province**: Dunhua City, Dehui City, Fuyu City (Jilin Provincial Forestry and Grassland Administration 2023).

**Liaoning Province**: Beipiao City, Shenyang City (field investigation) .

##### Notes

National Protected Plants (Level II).

#### Apiaceae

C3259A91-1123-5D3E-AE7F-C1BDBBBA77CF

#### Carlesia sinensis

Dunn

EB162A97-5AFF-5C67-BB91-937F33EC8184

##### Distribution

**Jilin Province**: Antu County, Changbai Mountain Nature Reserve (Jilin Provincial Forestry and Grassland Administration 2023).

**Liaoning Province**: Jianchang County, Chaoyang City, Fengcheng City, Dandong City, Donggang City, Anshan City, Jinzhou District, Zhuanghe City (Li et al. 2017).

##### Notes

National Protected Plants (Level II).

#### Glehnia littoralis

F. Schmidt ex Miq.

ABDA86DA-2A51-5311-99E7-6A2A6AADA15D

##### Distribution

**Liaoning Province**: Linghai City, Huludao City, Xingcheng City, Suizhong County, Gaizhou City, Wafangdian District, Pulandian District, Jinzhou District, Changhai County (Li 2019).

##### Notes

National Protected Plants (Level II).

#### Araliaceae

F9DBA85F-9E8A-5092-8E99-83C6CBE2ACCB

#### Panax ginseng

C. A. Mey.

06998E3A-5B8A-5B32-9D34-DF669CE6043B

##### Distribution

**Heilongjiang Province**: Shuangyashan City, Raohe County (Wang et al. 2022).

**Jilin Province**: Fusong County, Dunhua City, Jingyu County (Sun 2007, Liu 2014).

**Liaoning Province**: Tieling City, Qingyuan City, Xinbin County, Benxi City, Huanren County, Kuandian County, Fengcheng City, Anshan City, Yingkou City, Gaizhou City, Zhuanghe City (Wu 2012, Wang 2013, Liu 2019a).

##### Notes

National Protected Plants (Level II).

#### Aspleniaceae

564463CF-7F1A-57C8-ADC6-7C7225056911

#### Asplenium komarovii

Akasawa

A3C0C512-26FB-5644-80BD-88AA272614C2

##### Distribution

**Jilin Province**: Baishan City, Changbai Korean Autonomous County, Fusong County, Linjiang City, Ji'an City (Jia et al. 2023).

##### Notes

National Protected Plants (Level II).

#### Cabombaceae

2F68C9B6-5BB8-5EB8-A486-C75B27AEC4AE

#### Brasenia schreberi

J. F. Gmel.

4410F509-93D1-53D6-AF27-52DFA22B5D24

##### Conservation status

LC

##### Distribution

**Heilongjiang Province**: Yichun City (Yu 1991).

##### Notes

National Protected Plants (Level II).

#### Crassulaceae

FAEBE7B3-2143-5E96-9AD6-FC745B7EAB69

#### Rhodiola angusta

Nakai

76B9E925-CB51-5E69-B4D4-2B4309DC6CD3

##### Distribution

**Heilongjiang Province**: Harbin City, Shangzhi City (Wang et al. 2022).

**Jilin Province**: Antu County, Fusong County, Changbai Mountain Nature Reserve (Jia 2022).

##### Notes

National Protected Plants (Level II).

#### Rhodiola rosea

L.

350DCA71-00CF-50F0-A961-BAB046F58815

##### Distribution

**Heilongjiang Province**: Mudanjiang City (Wang et al. 2022).

**Jilin Province**: Antu County, Fusong County, Changbai Korean Autonomous County, Changbai Mountain Nature Reserve (Jilin Provincial Forestry and Grassland Administration 2023).

##### Notes

National Protected Plants (Level II).

#### Rhodiola sachalinensis

Boriss.

339E04BE-ABA2-5A8A-8201-D50F24932A25

##### Distribution

**Heilongjiang Province**: Harbin City, Mudanjiang City, Qiqihar City, Hailin, Ning'an City, Shangzhi City (Wang et al. 2022).

**Jilin Province**: Antu County, Fusong County, Changbai Korean Autonomous County, Linjiang City, Dunhua City, Changbai Mountain Nature Reserve (Sheng et al. 2004, Jilin Provincial Forestry and Grassland Administration 2023).

##### Notes

National Protected Plants (Level II).

#### Cupressaceae

C89D6DCE-3CE8-5F7B-B594-A6AF08F86965

#### Thuja koraiensis

Nakai

89B7B298-EBCD-52AD-9555-625D46124F13

##### Conservation status

VN

##### Distribution

**Jilin Province**: Antu County, Tonghua City, Baishan City, Yanbian Area, Changbai Mountain Nature Reserve (Lan et al. 2021, Jilin Provincial Forestry and Grassland Administration 2023).

##### Notes

National Protected Plants (Level II).

#### Droseraceae

4346291C-2D5E-515D-9F75-6D60B8CBB5FC

#### Aldrovanda vesiculosa

L.

E2BF7C17-2816-57A8-9A9E-A60697A9C75A

##### Conservation status

EN

##### Distribution

**Heilongjiang Province**: Qiqihar City, Tongjiang, Fuyuan (Shan et al. 2020, Wang et al. 2022).

##### Notes

National Protected Plants (Level I).

#### Ericaceae

F8D8A08B-4C56-5FED-AA8D-C466167541EA

#### Rhododendron dauricum

L.

932CB142-5E74-59CB-8F6E-9E60E17BE7CF

##### Distribution

**Heilongjiang Province**: Acheng District, Daxing'anling Area, Hailin City, Hegang City, Linkou County, Mohe City, Mudanjiang City, Qiqihar City, Shuangyashan City, Tahe County, Harbin City, Heihe City, Huma County, Hulin City, Jidong County, Jixi City, Jixian County, Jiayin County, Luobei County, Mishan City, Nenjiang City, Raohe County, Shangzhi City, Suifenhe City, Wuchang City, Yichun City (Wang et al. 2022).

**Jilin Province**: Linjiang City, Helong City, Antu County, Fusong County, Wangqing County, Hunchun City, Jiaohe City, Ji'an City, Yongji County, Huadian County, Jingyu City, Changbai Korean Autonomous County, Yanji City, Longjing City, Antu County, Changbai Mountain Nature Reserve (Man 2016, Du et al. 2019, Wu et al. 2023).

**Liaoning Province**: Beipiao City, Huanren County (Man 2016).

##### Notes

National Protected Plants (Level II).

#### Fabaceae

7914B78D-CE26-5AE1-B21D-A2869DBFFAAD

#### Glycine soja

Siebold & Zucc.

F718A20A-534A-501B-9C21-D3D305DC4362

##### Distribution

**Heilongjiang Province**: Counties of Heilongjiang Province (Liu 2004, Wei et al. 2011).

**Jilin Province**: Counties of Jilin Province (Liu 2004, Wei et al. 2011).

**Liaoning Province**: Counties of Liaoning Province (Liu 2004, Wei et al. 2011).

##### Notes

National Protected Plants (Level II).

(Fig. 2)

#### Glycyrrhiza uralensis

Fisch.

F40EDE9A-B527-523D-B473-4E2737DD8EA8

##### Distribution

**Heilongjiang Province**: Daqing City, Lindian County, Qiqihar City, Saertu District, Suihua City, Tailai County, Zhaoyuan County, Anda City, Duerbote Mongol Autonomous County, Zhaodong City, Zhaozhou County (Zhao et al. 2004, You et al. 2022, Wang et al. 2022).

**Jilin Province**: Qian'an County, Nong'an County, Qianguo'erluosi Mongolian Autonomous County, Da'an City, Fuyu City, Taonan City, Tongyu County, Zhenlai County, Changling County, Baicheng City, Songyuan City, Siping City, Yanbian Korean Autonomous Prefecture (Jilin Provincial Forestry and Grassland Administration 2023).

**Liaoning Province**: Jianping County, Beipiao City, Fuxin City, Heishan County, Zhangwu County, Kangping County (field investigation).

##### Notes

National Protected Plants (Level II).

#### Fucaceae

6231AB6B-1629-589B-859B-B4FD4DEC0FCD

#### Silvetia siliquosa

D47B7D49-12D6-5E61-8633-A554440908CC

##### Distribution

**Liaoning Province**: Liaoning coastal area, Changhai County, Jin County (Jinzhou District), Dalian City, Lushunkou District, Fu County (Wafangdian City) (Zeng and Zhang 1958).

##### Notes

National Protected Plants (Level II).

#### Haloragaceae

1197EBCD-04D9-5099-95A5-BCB98D1B2F99

#### Myriophyllum ussuriense

(Regel) Maxim.

B0AE11A3-40C2-515B-BC03-1B29944D7F8F

##### Conservation status

LC

##### Distribution

**Heilongjiang Province**: Heihe City, Qiqihar City, Luobei County, Hulin County, Mishan County, Raohe County, Jidong County, Nenjiang County, Nenjiang County, Boli County, Linkou County, Longjiang County, Fujin County, Huinan County, Fuyuan (Wang et al. 2022).

**Jilin Province**: Dunhua City, Helong City, Longjing City, Antu County (Jilin Provincial Forestry and Grassland Administration 2023).

##### Notes

National Protected Plants (Level II).

#### Hydrocharitaceae

F4C291B0-D995-5373-8BFC-68EE645AD396

#### Ottelia alismoides

(L.) Pers.

D43564C9-3413-5FD9-B8D3-56F2B305E14B

##### Conservation status

LC

##### Distribution

**Heilongjiang Province**: Hulin City, Jixi City, Qiqihar City, Mishan City (Wang et al. 2022).

##### Notes

National Protected Plants (Level II).

#### Liliaceae

F1161A5B-D780-54D9-B473-FA9C94806433

#### Fritillaria maximowiczii

Freyn

5EE9A4E4-24C1-57C1-89DA-87F54B6C2590

##### Distribution

**Heilongjiang Province**: Acheng District, Daxing'anling Prefecture, Qiqihar City, Yichun City, Huma County, Xunke County (Wang et al. 2022).

**Jilin Province**: Huinan County, Jingyu City, Fusong County, Changbai Korean Autonomous County, Liuhe County, Tonghua City, Jiaohe City, Dunhua City, Antu County (Jilin Provincial Forestry and Grassland Administration 2023).

**Liaoning Province**: Suizhong County, Jianchang County, Chaoyang City, Lingyuan City (field investigation).

##### Notes

National Protected Plants (Level II).

(Fig. 3)

#### Fritillaria usuriensis

Maxim.

5D80E406-54BC-56DF-B39E-E919C1D066CB

##### Distribution

**Heilongjiang Province**: Baoqing County, Harbin City, Heihe City, Hulin City, Mishan City, Ning'an City, Qiqihar City, Wuchang City, Yilan County, Shangzhi City, Yichun City (Wang et al. 2022).

**Jilin Province**: Dongchang District, Linjiang City, Jiutai District, Erdao District, Erdaojiang District, Nong'an County, Nanguan District, Shuangyang District, Antu County, Kuancheng District, Dehui City, Fusong County, Chaoyang District, Liuhe County, Huadian City, Meihekou City, Yushu City, Wangqing County, Lvyuan District, Shulan City, Jiaohe City, Huinan County, Tonghua County, Ji'an City (Li 2010, Jilin Provincial Forestry and Grassland Administration 2023).

**Liaoning Province**: Dandong City, Kuandian County, Fengcheng City, Benxi City, Huanren County, Qingyuan City, Xinbin County, Fushun City, Shenyang City (Li 2010).

##### Notes

National Protected Plants (Level II).

#### Lilium amabile

Palib.

38E05976-8B51-51D1-B042-FEEA83A6A0EA

##### Distribution

**Liaoning Province**: Dandong City, Fengcheng City, Donggang City, Shenyang City (Xu and Lei 2010, Cui 2023).

##### Notes

National Protected Plants (Level II).

#### Lycopodiaceae

2109D09A-D939-5279-879E-80A3AF09A9E9

#### Huperzia asiatica

(Ching) N. Shrestha & X. C. Zhang

80E9EAF4-AD93-530E-BC45-FE7C186F221E

##### Distribution

**Heilongjiang Province**: Harbin City, Ning'an City, Qiqihar City, Yichun City (Wang et al. 2022).

**Jilin Province**: Antu County, Wangqing County, Dunhua City, Changbai Mountain Nature Reserve (Jilin Provincial Forestry and Grassland Administration 2023).

##### Notes

National Protected Plants (Level II).

#### Huperzia miyoshiana

(Makino) Ching

C180496B-2D82-55B2-9983-4D9429294AB7

##### Distribution

**Heilongjiang Province**: Harbin City, Ning'an City, Qiqihar City, Yichun City (Wang et al. 2022).

**Jilin Province**: Linjiang City, Antu County, Fusong County, Changbai Korean Autonomous County, Jingyu County (Zhang and Kong 1998, Chinese Virtual Herbarium 2025, National Specimen Information Infrastructure 2025).

**Liaoning Province**: Kuandian County, Zhuanghe City (Chinese Virtual Herbarium 2025).

##### Notes

National Protected Plants (Level II).

#### Huperzia selago

(L.) Bernh. ex Schrank & Mart.

68070CEA-BB33-52E7-A329-AD3B421C0123

##### Distribution

**Heilongjiang Province**: Qitaihe City, Qiqihar City, Yichun City (Wang et al. 2022).

**Jilin Province**: Antu County, Fusong County, Changbai Korean Autonomous County, Jingyu County, Helong City (Zhang and Kong 1998).

##### Notes

National Protected Plants (Level II).

#### Huperzia serrata

(Thunb. ex Murray) Trevis.

7182147A-E935-5E6C-B73D-9B8E748BF4AF

##### Distribution

**Heilongjiang Province**: Dongning City, Hailin City, Shuangyashan City, Raohe County, Shangzhi City, Tahe County, Yichun City (Wang et al. 2011, Wang et al. 2022).

**Jilin Province**: Línjiāng City, Antu County, Fusong County, Dunhua City, Changbai Korean Autonomous County, Ji'an City, Jingyu County (Wang et al. 2011).

**Liaoning Province**: Benxi County, Kuandian County, Huanren County, Fengcheng City, Xinbin County (Wang et al. 2011).

##### Notes

National Protected Plants (Level II).

#### Lythraceae

DA7C3B2E-7CA8-57A2-8208-27B8620185B6

#### Trapa incisa

Siebold & Zucc.

3822A4DA-6DEE-5E25-8636-C86C745667BF

##### Conservation status

LC

##### Distribution

**Heilongjiang Province**: Harbin City, Qiqihar City, Shangzhi City (Wang et al. 2022).

**Jilin Province**: Fuyu City, Dehui City, Dunhua City, Hunchun City (Jilin Provincial Forestry and Grassland Administration 2023).

**Liaoning Province**: Kaiyuan County, Pulandian District (Lang and Zhang 1979).

##### Notes

National Protected Plants (Level II).

#### Malvaceae

08692EC4-EF46-5062-A637-0C7635719503

#### Tilia amurensis

Rupr.

FA1E54EE-4A62-59F5-B27E-D47BE00449E0

##### Conservation status

LC

##### Distribution

**Heilongjiang Province**: Acheng District, Binxian, Daqing City, Hulan District, Jidong County, Jixi City, Mudanjiang City, Mulan County, Nenjiang City, Qitaihe City, Qiqihar City, Shuangyashan City, Suihua City, Tonghe County, Anda City, Baoqing County, Bobo County, Harbin City, Heihe City, Luobei County, Mishan City, Ning'an City, Shangzhi City, Sunwu County, Yichun City (Zhou 2008, Wang et al. 2022).

**Jilin Province**: Dongchang District, Linjiang City, Erdaojiang District, Helong City, Antu County, Fusong County, Dunhua City, Liuhe County, Meihekou City, Wangqing County, Hunchun City, Jiaohe City, Huinan County, Tonghua County, Changbai Korean Autonomous County, Ji'an City, Jingyu County (Jilin Provincial Forestry and Grassland Administration 2023).

**Liaoning Province**: Xianrendong National Nature Reserve, Fengcheng City, Baishilazi National Nature Reserve, Xiuyan County, Kuandian County, Bailangshan National Nature Reserve, Fushun County, Xinbin County, Benxi County, Huanren County, Qingyuan County, Kaiyuan City, Tieling County, Xifeng County, Binglashan National Forest Park in various regions of Liaoning (field investigation) (Zhou 2008).

##### Notes

National Protected Plants (Level II).

#### Nymphaeaceae

7E92E64D-6B73-512F-A490-3812C1D3E6EF

#### Nelumbo nucifera

Gaertn.

FDB15029-523C-54D0-AA19-42A8BFE33C7A

##### Distribution

**Heilongjiang Province**: Sanjiang Plain, Songnen Plain, Fangzheng, Mulan County, Ning'an City, Wuchang City (Xue et al. 2005, Xue et al. 2015).

**Jilin Province**: Jingxin Town, Hunchun (Jilin Provincial Forestry and Grassland Administration 2023).

**Liaoning Province**: Huanren County, Jinzhou District, Pulandian District, Haicheng City, Liaoyang County, Liaozhong District, Xinmin City, Tai'an County, Suizhong County, Zhangwu County (field investigation) (Fu 2021, Plant photo bank of China 2025).

##### Notes

National Protected Plants (Level II).

(Fig. 4)

#### Oleaceae

963E058F-6EF1-5BCA-8556-7F999274D668

#### Fraxinus mandshurica

Rupr.

97E7D296-B832-5DBA-B64B-0563634AE801

##### Conservation status

LC

##### Distribution

**Heilongjiang Province**: Acheng District, Hegang City, Hulin City, Jixi City, Mishan City, Mudanjiang City, Qiqihar City, Harbin City, Yichun City (Zhao 2021, Wang et al. 2022, Liang et al. 2023).

**Jilin Province**: Dongchang District, Linjiang City, Erdaojiang District, Helong City, Antu County, Yanji City, Fusong County, Dunhua City, Liuhe County, Huadian City, Meihekou City, Yongji County, Wangqing County, Hunchun City, Panshi City, Shulan City, Jiaohe City, Huinan County, Tonghua County, Changbai Korean Autonomous County, Ji'an City, Jingyu County (Zhao 2021, Liang et al. 2023).

**Liaoning Province**: Haicheng City, Wafangdian District, Pulandian District, Zhuanghe City, Anshan City, Nanpiao District, Xifeng City, Benxi County, Dengta City, Fengcheng City, Fushun County, Gaizhou City, Ganjingzi District, Heishan County, Huanren County, Kuandian County, Mingshan District, Pingshan District, Dongling District, Qingyuan County, Shiqiaozhuang Development Zone, Suizhong County, Xinbin County, Xiuyan County, Yixian County (Zhao 2021, Liang et al. 2023).

##### Notes

National Protected Plants (Level II).

#### Orchidaceae

68E02505-8BB7-5D56-989C-90F44B46FCEB

#### Cypripedium calceolus

L.

7A650779-7F18-5335-BF0B-EC2B062BE2F1

##### Conservation status

LC

##### Distribution

**Heilongjiang Province**: Harbin City, Huma County, Jiayin County, Shangzhi City, Yichun City, Jixi Hulin Zhenbao Island Nature Reserve, Nenjiang Central Station Nature Reserve (Wang et al. 2022, Wu 2022).

**Jilin Province**: Linjiang City, Helong City, Antu County, Fusong County, Hunchun City, Huadian, Dunhua, Wangqing, Longjing, Tumen (Zhao 2013, Jilin Provincial Forestry and Grassland Administration 2023).

**Liaoning Province**: Benxi City, Huanren County, Qingyuan City, Xinbin County (field investigation) .

(Fig. 5)

##### Notes

National Protected Plants (Level II).

(Fig. 6)

#### Cypripedium guttatum

Sw.

FE1DB140-17D2-5C59-BB74-C80CFD3ADA8F

##### Conservation status

LC

##### Distribution

**Heilongjiang Province**: The Greater Khingan Range area, Harbin City, Hegang City, Hulin City, Jixi City, Qiqihar City, Tieli City, Heihe City, Huma County, Jiayin County, Shangzhi City, Yichun City, Raohe, Jiayin Maolan Gully, Heihe Shengshan Nature Reserve, Nenjiang Central Station Nature Reserve, Jiagedaqi Guli Forest Farm, Huzhong Nature Reserve (Li 2020, Wang et al. 2022, Wu 2022).

**Jilin Province**: Dongchang District, Linjiang City, Erdaojiang District, Helong City, Tumen City, Antu County, Yanji City, Fusong County, Dunhua City, Liuhe County, Meihekou City, Jiangyuan District, Wangqing County, Hunjiang District, Hunchun City, Huinan County, Tonghua County, Changbai Korean Autonomous County, Ji'an City, Jingyu County, Longjing City, Jiaohe, Yanbian, Changbai Mountain Nature Reserve (Zhao 2013, Jilin Provincial Forestry and Grassland Administration 2023).

**Liaoning Province**: Benxi City, Huanren County, Fengcheng City (Li 2020).

(Fig. 7)

##### Notes

National Protected Plants (Level II).

(Fig. 8)

#### Cypripedium macranthos

Sw.

79A5155F-02D7-53E4-BA36-975B91E6C92B

##### Conservation status

LC

##### Distribution

**Heilongjiang Province**: The Greater Khingan Range area, Harbin City, Hegang City, Heihe City, Huma County, Jixi City, Jiayin County, Luobei County, Mishan City, Ning'an City, Qiqihar City, Shangzhi City, Shuangyashan City, Yichun City, Raohe, Jixi Hulin Zhenbao Island Nature Reserve, Nenjiang Central Station Nature Reserve, Jiagedaqi Guli Forest Farm and Tahe (Li 2020, Wu 2022).

**Jilin Province**: Jiaohe City, Liuhe County, Jingyu City, Fusong County, Dunhua City, Wangqing County, Antu County, Yanji and Changbai Mountain Nature Reserve (Zhao 2013, Li 2020).

**Liaoning Province**: Xinbin County, Qingyuan City, Benxi City, Huanren County, Kuandian County, Dandong City, Fengcheng City, Kaiyuan City, Xifeng City (Li 2020).

(Fig. 9)

##### Notes

National Protected Plants (Level II).

(Fig. 10)

#### Cypripedium shanxiense

S. C. Chen

85CCADFA-9ED7-543C-9E75-2E727660464B

##### Conservation status

NT

##### Distribution

**Heilongjiang Province**: Ta River, Huzhong Nature Reserve, Yichun City (Wang et al. 2022).

**Jilin Province**: Baishan City, Tonghua City (Zhao 2013).

**Liaoning Province**: Huanren County (Li and Li 2020) .

(Fig. 11)

##### Notes

National Protected Plants (Level II).

#### Cypripedium × ventricosum

Sw.

67BDBBC6-8EF0-5D50-8CC3-BD6386A10AB4

##### Distribution

**Heilongjiang Province**: Yichun City, Jixi City, Jiamusi City (Wang et al. 2022, Wu 2022).

**Jilin Province**: Yanji City, Wangqing County, Jiaohe City, Hunchun City, Dunhua City, Longjing City, Antu County, Tumen City, Changbai Mountain Nature Reserve (Zhao 2013, Jilin Provincial Forestry and Grassland Administration 2023).

(Fig. 12)

##### Notes

National Protected Plants (Level II).

#### Gastrodia elata

Bl.

625B7E5A-9561-51DA-8309-0A0D2DB0858A

##### Conservation status

VN

##### Distribution

**Heilongjiang Province**: Xiaoxing'an Mountains, Laoyeling Mountains (Wang et al. 2022).

**Jilin Province**: Antu County (Jilin Provincial Forestry and Grassland Administration 2023).

**Liaoning Province**: Benxi City, Xinbin County, Huanren County, Kuandian County, Zhuanghe City, Xiuyan County (Guan 2006).

##### Notes

National Protected Plants (Level II).

(Fig. 13)

#### Gymnadenia conopsea

(L.) R. Br.

89F1467A-C392-5425-A28D-D043A4EA549B

##### Distribution

**Heilongjiang Province**: Huazhong, Xinlin District, Tahe Country, Jiagedaqi, Changbai Mountain, Huachuan County Xiaoshitou, Beian Weihuling, Qianshan, Xiaoxing'anling (Wang et al. 2022).

**Jilin Province**: Dongchang District, Linjiang City, Erdaojiang District, Antu County, Fusong County, Liuhe County, Huadian City, Meihekou City, Wangqing County, Hunchun City, Huinan County, Tonghua County, Changbai Korean Autonomous County, Ji'an City, Jingyu County, Jiaohe City, Dunhua City, Changbai Mountain Nature Reserve (Jilin Provincial Forestry and Grassland Administration 2023).

**Liaoning Province**: Qingyuan City, Huanren County, Fengcheng City, Xifeng City (National Specimen Information Infrastructure 2025).

##### Notes

National Protected Plants (Level II).

#### Orobanchaceae

1C16AC3B-71C1-555B-B2DC-08CBB0A20394

#### Boschniakia rossica

(Cham. & Schltdl.) B. Fedtsch.

9BEA0CF8-2E8E-5972-B24C-E6F0ED403691

##### Distribution

**Heilongjiang Province**: Huma County, Daxing'anling area (Sun et al. 1995, An et al. 2011).

**Jilin Province**: Antu County, Fusong County, Changbai Korean Autonomous County (Sun et al. 1995, Yu et al. 1996).

##### Notes

National Protected Plants (Level II).

(Fig. 14)

#### Pinaceae

1CEEFB12-0937-50CB-957B-1884D0646738

#### Pinus densiflora var. ussuriensis

Liou & Z. Wang

8235FEA3-A4AE-5628-95DA-AFBED090202A

##### Distribution

**Heilongjiang Province**: Jidong County, Mudanjiang City, Muling City, Jixi City, Mishan City (Chen and Sun 2012, Wang et al. 2022).

##### Notes

National Protected Plants (Level II).

#### Pinus koraiensis

Siebold & Zucc.

A8BCEFFE-41AE-5E97-945B-51D0409757EA

##### Conservation status

LC

##### Distribution

**Heilongjiang Province**: Dongning City, Hailin City, Hegang City, Hulin City, Jidong County, Jixi City, Jiamusi City, Mishan City, Mudanjiang City, Ning'an City, Shangzhi City, Shuangyashan City, Suifenhe City, Suihua City, Tieli City, Xinlin District, Raohe County, Tangyuan County, Yichun City (Wu and Han 1990, Jia 2011, Song 2013, Dong 2018, Chen et al. 2021, Wang et al. 2022).

**Jilin Province**: Dongchang District, Linjiang City, Jiutai District, Erdaojiang District, Helong City, Antu County, Fusong County, Dunhua City, Liuhe County, Huadian City, Meihekou City, Wangqing County, Jiaohe City, Huinan County, Tonghua County, Changbai Korean Autonomous County, Ji'an City, Jingyu County (Wang 2007, Jia 2011, Shang et al. 2013, Dong 2018, Abudureheman et al. 2023).

**Liaoning Province**: Benxi County, Huanren County, Fengcheng City, Kuandian County, Xinbin County (Guo et al. 1998, Yao 2019).

##### Notes

National Protected Plants (Level II).

#### Pinus sylvestris var. sylvestriformis

(Taken.) W. C. Cheng & C. D. Chu

6FEC328A-31A1-5A63-A190-28774FBF89E5

##### Distribution

**Jilin Province**: Dongchang District, Jiutai District, Erdaojiang District, Helong City, Antu County, Fusong County, Dunhua City, Liuhe County, Meihekou City, Wangqing County, Jiaohe City, Huinan County, Tonghua County, Changbai Korean Autonomous County, Ji'an City (Jilin Provincial Forestry and Grassland Administration 2023).

##### Notes

National Protected Plants (Level II).

#### Poaceae

B6AC681C-DB8D-5BDF-9123-100D2D2561C1

#### Coleanthus subtilis

(Tratt.) Seidel

0CCB0D1D-B05A-5D19-A413-E73ACA63BAF6

##### Conservation status

LC

##### Distribution

**Heilongjiang Province**: Harbin City (Wang et al. 2022).

##### Notes

National Protected Plants (Level II).

#### Zoysia sinica

Hance

B4A58672-8C8D-5B6D-98C6-F55B7E31900D

##### Distribution

**Liaoning Province**: Dalian suburbs (Heishan County), Pulandian District (Chengzi Tan) (Wang 2003).

##### Notes

National Protected Plants (Level II).

#### Rosaceae

EAE349A2-548B-5DA0-89F8-AD4F165436AA

#### Malus komarovii

(Sarg.) Rehder

D312682D-74DA-5636-9168-35A0717F2B46

##### Conservation status

EN

##### Distribution

**Jilin Province**: Antu County, Changbai Korean Autonomous County, Baishan City, Linjiang City, Changbai Mountain Nature Reserve (Jilin Provincial Forestry and Grassland Administration 2023).

##### Notes

National Protected Plants (Level II).

#### Rosa rugosa

Thunb.

2C0A8239-02E5-5808-BE8A-93A84B5CECBE

##### Distribution

**Jilin Province**: Hunchun Jingxin Town (Zhao 2008).

**Liaoning Province**: Jinzhou District, Changhai County, Lushunkou District, Zhuanghe City, Dalian City, Donggang City, Yingkou Bayuquan (Qin et al. 1994, Zhao 2008).

##### Notes

National Protected Plants (Level II).

(Fig. 15)

#### Rutaceae

8898F9CE-857E-563D-84BD-93E929059D3D

#### Phellodendron amurense

Rupr.

AA17F1D1-4BD4-5620-8ACC-27C7D3EF0A9E

##### Distribution

**Heilongjiang Province**: Bin County, Bobl County, Daqing Mountain County, Daxing'anling Area, Dongning City, Fujin City, Hegang City, Huma County, Hulin City, Huachuan County, Jixi City, Jiayin County, Linkou County, Luobei County, Mudanjiang City, Mulan County, Muling City, Ning'an City, Qiqihar City, Qing'an County, Raohe County, Shuangyashan City, Suiling County, Sunwu County, Tangyuan County, Tonghe County, Wuchang City, Xunke County, Yanshou County, Yilan County, Baoqing County, Harbin City, Heihe City, Jixian County, Mishan City, Nenjiang City, Shangzhi City, Yichun City (Yan et al. 2006, Zhang et al. 2012, Zhang et al. 2016, Wang et al. 2022).

**Jilin Province**: Dongchang District, Linjiang City, Jiutai District, Erdaojiang District, Helong City, Antu County, Yanji City, Fusong County, Dunhua City, Liuhe County, Huadian City, Meihekou City, Yongji County, Wangqing County, Hunchun City, Panshi City, Shulan City, Jiaohe City, Huinan County, Tonghua County, Changbai Korean Autonomous County, Ji'an City, Jingyu County (Yan et al. 2006, Xian 2011, Yi et al. 2013, Zhang 2016).

**Liaoning Province**: Beizhen City, Benxi County, Dengta City, Fengcheng City, Fushun County, Gaizhou City, Haicheng City, Huanren County, Kaiyuan City, Kuandian County, Lianshan District, Liaoyang County, Mingshan District, Nanfen District, Qinghe District, Qingyuan County, Shuncheng District, Suizhong County, Tieling County, Xifeng County, Xinbin County, Xiuyan County, Zhen'an District (Yan et al. 2006, Zhang 2016, Gu 2018, Zhang 2019).

##### Notes

National Protected Plants (Level II).

(Fig. 16)

#### Scheuchzeriaceae

65EB85F6-AD48-5B26-80FE-7498C5C96926

#### Scheuchzeria palustris

L.

F9C53B40-FADC-5252-8D8C-18975F9C8074

##### Conservation status

LC

##### Distribution

**Heilongjiang Province**: Suifenhe City, Hailin City, Ning'an City, Muling City, Dongning County, Linkou County, Jixi City, Hulin City, Mishan City, Jidong County, Shuangyashan City, Jixian County, Youyi County, Baoqing County, Raohe County (Wang et al. 2022).

**Jilin Province**: Linjiang City, Changbai and Changbai Mountain Nature Reserve (Jilin Provincial Forestry and Grassland Administration 2023, National Specimen Information Infrastructure 2025).

##### Notes

National Protected Plants (Level II).

#### Sphagnaceae

FB0D7E04-1296-53D1-AD96-00F5D1ED60EE

#### Sphagnum multifibrosum

X. J. Li & M. Zang

83AC57CB-B578-570C-A080-CD8FECE2770D

##### Distribution

**Heilongjiang Province**: The Lesser Khingan Range (accessed at the Herbarium of the Shenyang Institute of Ecology).

##### Notes

National Protected Plants (Level II).

#### Sphagnum squarrosum

Crome

EDF7314B-ADF4-5495-B23A-1F1B64580063

##### Distribution

**Heilongjiang Province**: The forestry bureaus of Xiaoxing'anling such as Hongxing, Fenglin, Shuangzi River, Cuiluan, Dailing etc., the forestry bureaus of Daxing'anling such as Mo'erga, Genhe and A'ershan etc., the forest areas of Ning'an County including the Daha Lin and Jingpo Lake, as well as Yichun, Ningan, Huma County, Longjiang County and Hailin City (accessed at the Herbarium of the Shenyang Institute of Ecology).

**Jilin Province**: Jiaohe County, Antu County, Dunhua County, Fusong County, Linjiang County, Changbai County, Jingyu City, Changbai Mountain Nature Reserve (accessed at the Herbarium of the Shenyang Institute of Ecology).

##### Notes

National Protected Plants (Level II).

#### Taxaceae

19AD249F-1E6A-5438-901E-7FF6245D2C72

#### Taxus cuspidata

Siebold & Zucc.

7CECA30F-0834-5112-9968-0AAA4168C1A0

##### Conservation status

LC

##### Distribution

**Heilongjiang Province**: Mudanjiang City, Muling City, Suileng County, Ning'an City (Wang et al. 2022).

**Jilin Province**: Linjiang City, Helong City, Antu County, Fusong County, Dunhua City, Wangqing County, Changbai Korean Autonomous County, Jingyu County (Jilin Provincial Forestry and Grassland Administration 2023).

**Liaoning Province**: Benxi County, Huanren County, Kuandian County (Liu 2007).

(Fig. 17)

##### Notes

National Protected Plants (Level I).

#### Tricholomataceae

A3B576B7-72CD-5ABB-B24C-DC54185C432C

#### Tricholoma matsutake

(S. Ito & S. Imai) Singer

B7E4D1EA-A138-56FE-B912-B73C8A25C141

##### Distribution

**Heilongjiang Province**: Dongning City (Wang et al. 2022).

**Jilin Province**: Jilin City, Yanbian Korean Autonomous Prefecture, Tonghua County, Baishan City, the eight counties and cities of Yanbian Prefecture, Helong City, Longjing City, Wangqing County, Antu County (Xu 2021).

**Liaoning Province**: Kuandian County (field investigation).

##### Notes

National Protected Plants (Level II).

#### Typhaceae

16CC69F8-B37D-5608-BD71-B21EF5698E17

#### Sparganium hyperboreum

Laest. ex Beurl.

E0269C5C-5422-5011-B832-3E7AF77D1020

##### Conservation status

LC

##### Distribution

**Heilongjiang Province**: Harbin City, Yichun City, Muling Country, Mohe County (Wang et al. 2022).

**Jilin Province**: Heilong City, Antu County (Jilin Provincial Forestry and Grassland Administration 2023).

##### Notes

National Protected Plants (Level II).

## Analysis

### Distribution at the county level

**Figure 18.**
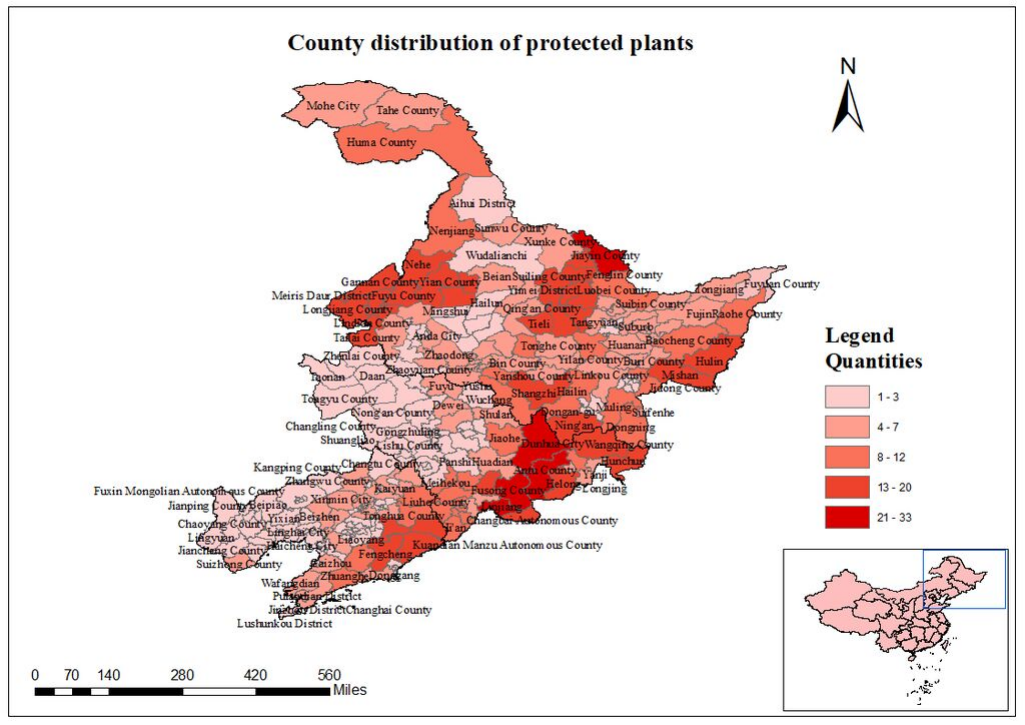
The number of species of nationally protected key plants at the county level in the three most north-eastern provinces.

The three most north-eastern provinces of China (Heilongjiang, Jilin and Liaoning) have a total of 34 prefecture-level cities, one region and one autonomous prefecture, along with 275 counties (cities, districts). In Jilin Province, the protected plant species in Antu County and Fusong County exceed 20 species. Amongst these, Antu County has the highest number of protected plant species, with 30 species, followed by Fusong County, which has 27 species. In total, 30 counties (cities, districts) have between 16 to 20 species of nationally protected plants, accounting for 10.91% of the total counties (cities, districts). Of these, 27 are located in Heilongjiang Province, two in Jilin Province and one in Liaoning Province. There are 32 counties (cities, districts) with 11 to 15 species of nationally protected plants, making up 11.64% of the total. Of these, 15 are in Heilongjiang Province, 13 in Jilin Province and four in Liaoning Province. Thirty-five counties (cities, districts) have between 6 and 10 species of nationally protected plants, accounting for 12.73% of the total. Amongst these, 19 are in Heilongjiang Province, six in Jilin Province and 10 in Liaoning Province. The vast majority of counties (cities, districts) have 1 - 5 species of nationally protected plants, accounting for 64% of the total counties (cities, districts) (Fig. 18).

### Distribution by geographical units

**Figure 19.**
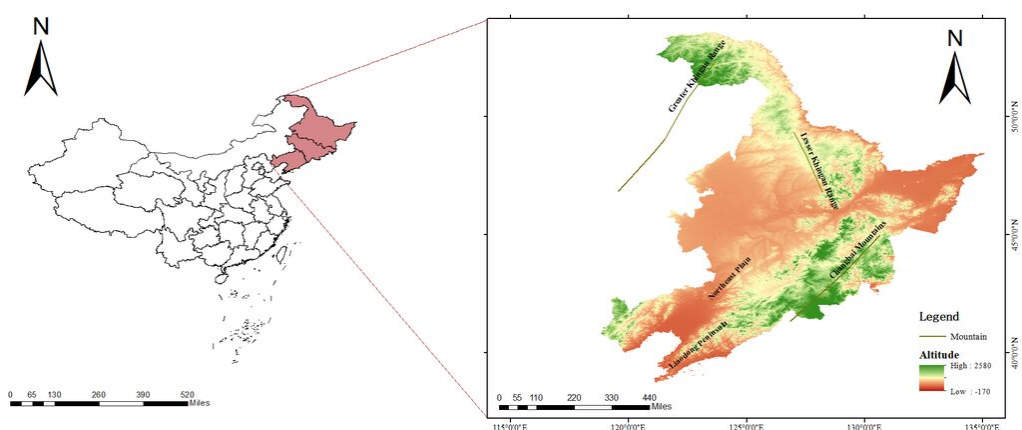
The terrain and mountain ranges in the three most north-eastern provinces.

The three most north-eastern provinces of China (Heilongjiang, Jilin and Liaoning) can be divided into several geographical regions: the Greater Khingan Range, the Lesser Khingan Range, the Changbai Mountains, the Northeast Plain and the Liaodong Peninsula (Fig. 19). The Greater Khingan Range is home to nine families, 10 genera and 13 species of protected plants. The Lesser Khingan Range is home to 12 families, 14 genera and 20 species of protected plants.The Changbai Mountains consist of a series of parallel northeast-southwest mountain ranges and several northwest- and east-west-orientated mountain ranges. The major mountains include the Zhangguangcai Ridge, Xialaoye Ridge, Jilin Hadaling, Weihuling, Longgang Mountain, Laoling, Mudan Ridge, Ying'e Ridge, Zengfeng Ridge, Nangang Mountain, Harbaling, Laosong Ridge, Mulingwoji Ridge, Panling and Dalong Ridge. The Changbai Mountains host 26 families, 29 genera and 39 species of protected plants. Amongst these, the Zhangguangcai Ridge has the highest number of protected plant species, with 23 families, 27 genera and 34 species. The Mudan Ridge and Harbaling both have 20 families, 24 genera and 30 species. Laoling has 18 families, 21 genera and 29 species.The Northeast Plain is composed of the Songnen Plain, the Sanjiang Plain and the Liaohe Plain. The Songnen Plain hosts 22 families, 24 genera and 30 species of protected plants. The Sanjiang Plain is home to 15 families, 16 genera and 21 species. The Liaohe Plain has 12 families, 14 genera and 14 species of protected plants. The Liaodong Peninsula, located in the south-eastern part of Liaoning Province, has 16 families, 20 genera and 22 species of protected plants.

**Figure 20.**
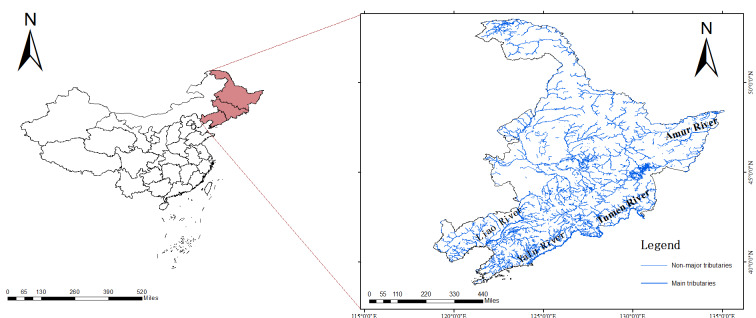
River map of the three most north-eastern provinces.

The region can be divided into four river basins: the Heilong River (Amur River), the Liao River, the Yalu River and the Tumen River (Fig. 20).The Heilong River is divided into three tiers of tributaries. The first-tier tributaries flowing through the three most north-eastern provinces are Beijicun River, Emuer River, Pangu River, Xiergenqi River, Huma River, Kuan River, Tuoniu River, Fabila River, Cierbin River, Shijin River, Gongbiela River, Xun River, Kuerbin River, Wuyun River, Jielie River, Ulagu River, Jiayin River, Yadan River, Lianhua River, Qinglong River, Yalu River, Nongjiang River, Songhua River, Ussuri River and Hutong River. The second-tier tributaries flowing through the three provinces include the Emuer River tributary, Huma River tributary, Xun River tributary, Kuerbin River tributary, Songhua River tributary and Ussuri River tributary. The third-tier direct tributaries include Hama River, Songmu River, Yichun River and Qixing River. Amongst these, the Songhua River is the largest first-tier tributary of the Heilong River, with its tributaries including the Nen River, Majiagou River, Mudan River and Hulan River, hosting 18 families, 21 genera and 27 species of protected plants. The Ussuri River is the second-largest, with tributaries such as Songacha River, Xiaomuling River, Muling River and Qihulin River, hosting 13 families, 13 genera and 15 species of protected plants.The Liao River flowing through the three most north-eastern provinces has major tributaries including the West Liao River, East Liao River, Xiushui River, Raoyang River, Zhaosutai River, Yangximu River and Chai River, with 10 families, 12 genera and 12 species of protected plants.The Yalu River, which also flows through the three most north-eastern provinces, has major tributaries such as the Hun River, Pushi River and the Yuan River, hosting 15 families, 17 genera and 23 species of protected plants.The Tumen River flows from south to north through four counties and cities in China: Helong, Longjing, Tumen and Hunchun. Its main tributaries are located within these counties and cities, with 12 families, 13 genera and 15 species of protected plants.

### Threat level

**Figure 21.**
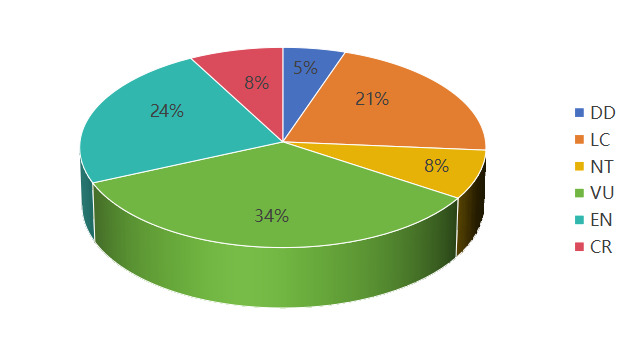
Threat level of national key protected plants in the three most north-eastern provinces in China.

According to the *China Biodiversity Red List – Higher Plants Volume (2020)*, amongst the nationally protected plants in the three most north-eastern provinces, there are two species with insufficient data (DD), eight species classified as Least Concern (LC), three species classified as Near Threatened (NT), thirteen species classified as Vulnerable (VU), nine species classified as Endangered (EN) and three species classified as Critically Endangered (CR). The species classified as Vulnerable (VU), Endangered (EN) and Critically Endangered (CR) are collectively referred to as "threatened species". There are 25 threatened species in total, accounting for 65.79% of the total number (Fig. 21).

**Figure 22.**
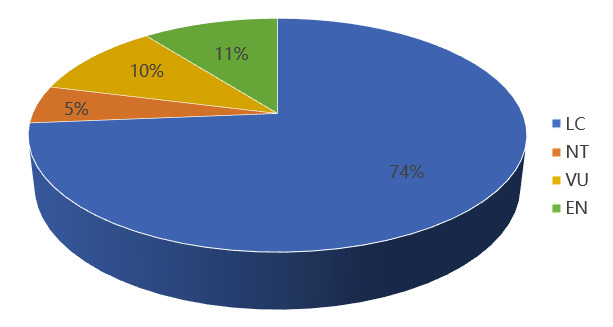
Global threat level level of national key protected plants in the three most north-eastern provinces.

According to the International Union for Conservation of Nature (IUCN 2002), there are 19 nationally protected plants in the three most north-eastern provinces that have been assessed and have sufficient data. Amongst these, 14 species are classified as Least Concern (LC), one species is classified as Near Threatened (NT), two species are classified as Vulnerable (VU) and two species are classified as Endangered (EN) (Fig. 22).

### Threat factors

According to the standards of the International Union for Conservation of Nature (IUCN), the factors that lead to the loss of biodiversity are categorised into 12 types: (1) Residential & commercial development; (2) Agriculture & aquaculture; (3) Energy production & mining; (4) Transportation & service corridors; (5) Biological resources use; (6) Human intrusions & disturbance; (7) Natural system modifications; (8) Invasive and other problematic species, genes & diseases; (9) Pollution; (10) Geological events; (11) Climate change & severe weather; (12) Other factors. Amongst these, 10 threats affect the key protected plant species in the north-eastern provinces of China, namely: Residential & commercial development; Agriculture & aquaculture; Energy production & mining; Transportation & service corridors; Biological resources use; Human intrusions & disturbance; Natural system modifications; Invasive and other problematic species, genes & diseases; Pollution; Climate change & severe weather. There are seven plants with unknown threat factors, including *Braseniaschreberi*, *Trapaincisa*, *Myriophyllumussuriense*, *Coleanthussubtilis*, *Sparganiumhyperboreum*, *Gastrodiaelata* and *Scheuchzeriapalustris* (Table 1).

### Current status of research

Amongst the 51 nationally protected plants, the more researched species were mostly those with economic value, such as *Pinuskoraiensis*, *Tricholomamatsutake*, *Actinidiaarguta*, *Nelumbonucifera*, *Glycinesoja* and *Panaxginseng*. There are seven species that are basically not covered by research, including *Cypripediumcalceolus*, Cypripedium×ventricosum, *Huperziamiyoshiana*, *Huperziaselago*, *Huperziaasiatica*, *Sparganiumhyperboreum* and *Myriophyllumussuriense*, five of which are in the new version of the List of Wild Plants under State Key Conservation (2021) (National Forestry and Grassland Administration 2021) and the current status of the resources of *Sparganiumhyperboreum* and *Myriophyllumussuriense* are still not clear.

Most of the nationally protected plants in the three most north-eastern provinces of China have medicinal, edible, industrial and ornamental values. Amongst them, *Aldrovandavesiculosa* can be used as an environmental indicator plant and its presence signals that the ecological environment of the watershed is excellent (Shan et al. 2020). In addition to its edible and ornamental values, *Maluskomarovii* can be widely used as an excellent rootstock for breeding new cold-resistant dwarf varieties of the genus Malus (Wang et al. 2022). *Thujakoraiensis* has important academic research value for exploring the ancient flora and cypress family classification and phylogeny (Yuan et al. 2022).

Most plants reproduce in two ways, sexual and asexual. Sexual reproduction is mainly through seeds and asexual reproduction is mainly through nutrient organs. *Boschniakiarossica* is a highly-specialised parasitic plant that only parasitises the roots of *Alnusmandshurica* or *Alnushirsuta* of the genus *Alnus* in the family Betulaceae and requires seeds to be able to access the host root tip in order to reproduce (Chen et al. 2015). Some scholars have found that *Cypripediummacranthos* is in highly-specialised symbiosis with Epulorhiza, which has an obvious promotion effect on *Cypripediummacranthos* seed germination. It has been shown that the most effective propagation method for non-symbiotic germination of *Cypripediummacranthos* is isolated seed germination, which is also a commonly used method of artificial propagation in orchids. It has been found that *Cypripediummacranthos* can also rely on other mycorrhizal fungi for the nutrients needed for seed germination and growth (Fan et al. 2023). It is now understood that *Armillariamellea* is a symbiotic species of *Gastrodiaelata*, which can only grow if *Armillariamellea* provides it with nutrients and the two are interdependent. Research has shown that the more vigorous growth of *Armillariamellea*, the more nutrition for *Gastrodiaelata*, the more rapid growth. *Gastrodiaelata* seedling reproduction methods are divided into sexual reproduction and asexual reproduction of two kinds. Sexual reproduction is propagation with *Gastrodiaelata* seeds, which requires seeds, germinating bacteria and *Armillariamellea*; asexual propagation takes nutrient organs, i.e. rice or white flax as propagation materials, which requires rice or white flax and *Armillariamellea* (*Wang 2023, You 2023*).

According to the literature examined, it is known that in-situ protection has been adopted for *Thujakoraiensis* and a nature reserve for *Thujakoraiensis* has been established in Changbai Mountain, China (Zhou et al. 2023). It has been analysed and found that more than half of the Orchidaceae in China have been protected in situ to varying degrees in protected areas (Li 2020). It is now understood that in-situ conservation is one of the most important measures to address the conservation, restoration and reconstruction of *Liliumamabile* populations (Cui 2023).

A total of 51 species of national key protected wild plants, belonging to 38 genera and 31 families, are found in the three north-eastern provinces of China. Of these, 72.55% are distributed within 77 national nature reserves in the region, primarily concentrated in the Changbai Mountain National Nature Reserve, Liangshui National Nature Reserve and Bailang Mountain National Nature Reserve. The majority of these protected plants are conserved in situ. However, 14 species have not been found within any national nature reserves, including *Scheuchzeriapalustris*, *Zoysiasinica*, *Coleanthussubtilis*, *Rhodiolasachalinensis*, *Cypripediumshanxiense*, *Silvetiasiliquosa*, *Sphagnummultifibrosum*, *Trapaincisa*, *Glehnialittoralis*, *Huperziamiyoshiana*, *Huperziaselago*, *Huperziaserrata*, *Huperziaasiatica* and *Otteliaalismoides*. Amongst them, *Scheuchzeriapalustris*, *Sphagnummultifibrosum*, *Huperziamiyoshiana*, *Huperziaselago* and *Huperziaasiatica* are data deficient, indicating significant research gaps.Research has been conducted on the geographical distribution and endangered status of *Coleanthussubtilis* both in China and globally (Liu et al. 2022); on seedling collection and propagation techniques, active ingredient extraction and fibre preparation of *Silvetiasiliquosa* (Huang et al. 2008, Kui et al. 2023); on the geographical distribution patterns, morphological diversity, physicochemical properties and chromosome-level genome of *Trapaincisa* (Guan et al. 2009, Xue et al. 2016, Qu et al. 2023); on the new geographical distribution, endophytic fungal species, seed morphology and germination characteristics of *Cypripediumshanxiense* (Gu et al. 2018, Li and Li 2020, Zhou et al. 2024); on photosynthesis, carbon concentration mechanisms, fruit and seed developmental morphology of *Otteliaalismoides* (*Fu et al. 2018*); on chemical composition, cultivation techniques and field re-introduction of *Rhodiolasachalinensis* (Liu et al. 2015, Jia 2022); on physiological and biochemical characteristics, reproduction techniques, applications and habitat protection of *Zoysiasinica* (Du et al. 2007, Ma 2011, Zhang 2014); on physiological and biochemical properties, chemical composition, application value, genetic diversity, anatomy, breeding systems and embryology of *Glehnialittoralis* (Yang et al. 2023, Zhou 2023, An et al. 2024); and on physiological and biochemical properties, habitat suitability prediction, reproduction, value, cultivation techniques, population structure and threat factors of *Huperziaserrata* (Ma et al. 2016, Qi and Wang 2017, Chen et al. 2022, Chen and Yu 2023, Pang 2023). While research progress on these plants has been made in certain areas, there are still significant research gaps.

## Discussion

### Distribution pattern

The distribution of national key protected plants in the three most north-eastern provinces shows obvious regional differences. Heilongjiang Province has the largest number of protected plant species and dominates the area, which may be related to its extensive forest cover and diverse ecosystems (Dong et al. 1988). Although Jilin Province is smaller in size, it has a more concentrated distribution of protected plants, especially in the Changbai Mountain area, reflecting the importance of the region in ecological conservation (Jilin Provincial Department of Ecology and Environment 2024). Liaoning Province has a relatively low distribution of protected plants, which may be related to its higher level of urbanisation and intensity of human activities (Lu et al. 2022). The Changbai Mountain Range is the region with the most concentrated distribution of protected plants in the three north-eastern provinces, especially Zhangguangcailing, Mudanling and Harbaling, reflecting the importance of this region in biodiversity conservation (Duan et al. 2019). Daxing'anling is the northernmost mountain range in China, with a cold climate and vegetation dominated by cold-temperate coniferous forests, with relatively few protected plant species, but with unique ecological value (Shu et al. 2024). The greater number of protected plant species in the Xiaoxinganling reflects the complexity and stability of its ecosystem (Liu 2012). The distribution of protected plants in the Northeast Plain is greatly affected by agricultural development and urbanisation, especially in the Liaohe Plain, where there are fewer protected plant species (Liu et al. 2024). The Liaodong Peninsula has more protected plant species, but the urbanisation process may pose a threat to its ecosystem (Xu 2024). The Songhua River Basin is the area with the most concentrated distribution of protected plants, reflecting its importance in the ecosystems of the three north-eastern provinces (PRC Ministry of Ecology and Environment et al. 2018). The Yalu River Basin has a large number of protected plant species, reflecting the complexity and stability of its ecosystem (Cao et al. 2006). The Liao River Basin has a low distribution of protected plants, which may be related to the high level of agricultural development and urbanisation in the Basin (Li et al. 2022). The distribution of protected plants in the Tumen River Basin is more uniform, but with fewer species, which may be related to the smaller size of the Basin and the intensity of human activities (Wang et al. 2002). In summary, the distribution of protected plants in the three most north-eastern provinces is influenced by a variety of factors, including geography, climate, ecosystem type and human activities.

### Endangered status and research status

The results of these assessments reflect the current conservation status and the level of threat faced by plants under State Key Conservation in the three north-eastern provinces. The threat factors for some plants are not yet clear, including *Braseniaschreberi*, *Trapaincisa*, *Myriophyllumussuriense*, *Otteliaalismoides*, *Coleanthussubtilis*, *Sparganiumhyperboreum*, *Gastrodiaelata* and *Scheuchzeriapalustris*. Therefore, in-depth ecological studies on these plants are urgently needed to clarify the specific threat factors they face and to provide a scientific basis for formulating effective conservation measures.

Progress has been made in scientific research and conservation of national key protected plants in the three north-eastern provinces, but some research gaps and conservation challenges remain. For example, the *Cypripediumshanxiense*, as a national key wild plant under Grade II protection, is listed as a vulnerable species (VU) in the Red List of Chinese Species and in Appendix II of the Convention on International Trade in Endangered Species of Wild Fauna and Flora (CITES) and its trade needs to be strictly regulated in order to avoid impacts on its survival (Import and Export Administration Office of the People's Republic of China for Endangered Species and Scientific Committee on Endangered Species of the People's Republic of China 2010). However, some of the plants are still understudied and the current status of the resources is unknown. For example, relatively little research has been done on plants such as *Huperziamiyoshiana*, *Huperziaselago*, *Huperzialucidula*, *Myriophyllumussuriense* and *Sparganiumhyperboreum*. Amongst them, although the *Myriophyllumussuriense* and *Sparganiumhyperboreum* are listed in the new version of the List of Wild Plants under State Key Protection in 2021, the status of their resources is still not fully clarified. Orchidaceae have important scientific research value due to their unique morphological features and ecological habits. However, rampant illegal collection and trade, coupled with their demanding habitat requirements, have led to a serious endangerment of their wild resources. The 2021's newly-adjusted List of Wild Plants under State Key Protection included 349 species of Orchidaceae from 23 genera and more than 1,000 species of Orchidaceae are now located in national nature reserves. Conservation measures for Orchidaceae include mapping the resource base, carrying out science popularisation and education and strengthening artificial breeding and cultivation (Zhang et al. 2022).

Overall, research on plants under national key protection in the three most north-eastern provinces has achieved certain results and relevant monographs, such as the Atlas of Plant Distribution in Northeast China (Cao 2019a), Atlas of the Primary Colours of Forest Plants in Northeast China (Cao 2019b) and Atlas of Major Forest Plants and Their Anatomy in Northeast China (Wang et al. 2018), have provided important references for plant research. However, there are still some plants that lack systematic research and conservation measures, which urgently need more attention. In the future, we should further deepen the research on plants under national key protection and improve conservation strategies to ensure their survival and reproduction.

### Limitations and future research

This study mainly collects information on plants under national priority protection through online databases, literature and some field survey data. Although a large amount of relevant data is covered, there are still problems of incomplete or untimely updating of data.

Firstly, limitations in data sources may affect the accuracy of the study results. Some of the studies relied on historical documents or fragmented data records and, although they combined field surveys, they were limited in scope and failed to cover the distribution areas of all protected plants due to time and resource constraints, resulting in insufficiently current and comprehensive data. In addition, the resource status of certain plants is still unclear, such as *Myriophyllumussuriense* and *Sparganiumhyperboreum*, which are included in the list of national key wild plants for protection, but lack detailed data on the current status of the resources and systematic assessment of the threatened factors.

Secondly, the endangered status has dynamic changes and uncertainty. This study categorises the threat factors of plants under national key protection in the three most north-eastern provinces, based on IUCN standards, but these threat factors may change over time due to environmental changes and human activities. For example, climate change, land-use change and increased human disturbance may further deteriorate the survival of certain species or their threat level may decrease after effective conservation measures are taken. Continuous monitoring and dynamic assessment are therefore essential.

In addition, there are limitations and research gaps in the data on conservation measures. This paper mainly counted in-situ conservation, focusing on the distribution of protected plants in national nature reserves. However, the three key conservation measures of relocation protection, field return and isolated conservation were not addressed, resulting in certain data gaps in conservation measures. For example, many rare and endangered plants have been relocated and bred in botanical gardens, scientific research institutes or specialised conservation bases, but there are fewer systematic assessments of their growth status, survival rate and reproductive capacity in relocated environments. In addition, there is still a lack of relevant experimental data on key protected plants in the three most north-eastern provinces for field return as an important measure to restore populations of endangered species. In-vitro conservation techniques, such as seed banking, tissue culture and cryopreservation, are essential for the long-term conservation of genetic resources, but there are fewer studies on their application to protected plants in the three most north-eastern provinces.

Based on the above limitations, future research should be carried out in the following aspects: (1) Strengthening data collection and updating: using modern scientific and technological means such as remote sensing technology, GIS spatial analysis and drone monitoring, combined with data from long-term field surveys, systematic surveys of protected plant resources, especially for species with a lack of data and long-term ecological monitoring and resource surveys should be carried out in order to fill in the gaps in research. At the same time, the monitoring and survey data will be integrated into a database and updated and shared in a timely manner; (2) Evaluation and monitoring of endangered status: For the national key protected plants in different areas of the three north-eastern provinces, carry out evaluation of climate change, habitat destruction, human interference and other influencing factors and establish a long-term monitoring mechanism; (3) Strengthening research on conservation measures: Investigations on relocation breeding in botanical gardens, scientific research institutes or specialised conservation bases should be strengthened and technical research on relocation conservation and isolated conservation should be enhanced for plants under national key protection outside nature reserves, as well as exploring suitable strategies for return to the wild to improve the ability of populations to survive.

## Supplementary Material

XML Treatment for Actinidiaceae

XML Treatment for Actinidia arguta

XML Treatment for Alismataceae

XML Treatment for Sagittaria natans

XML Treatment for Apiaceae

XML Treatment for Carlesia sinensis

XML Treatment for Glehnia littoralis

XML Treatment for Araliaceae

XML Treatment for Panax ginseng

XML Treatment for Aspleniaceae

XML Treatment for Asplenium komarovii

XML Treatment for Cabombaceae

XML Treatment for Brasenia schreberi

XML Treatment for Crassulaceae

XML Treatment for Rhodiola angusta

XML Treatment for Rhodiola rosea

XML Treatment for Rhodiola sachalinensis

XML Treatment for Cupressaceae

XML Treatment for Thuja koraiensis

XML Treatment for Droseraceae

XML Treatment for Aldrovanda vesiculosa

XML Treatment for Ericaceae

XML Treatment for Rhododendron dauricum

XML Treatment for Fabaceae

XML Treatment for Glycine soja

XML Treatment for Glycyrrhiza uralensis

XML Treatment for Fucaceae

XML Treatment for Silvetia siliquosa

XML Treatment for Haloragaceae

XML Treatment for Myriophyllum ussuriense

XML Treatment for Hydrocharitaceae

XML Treatment for Ottelia alismoides

XML Treatment for Liliaceae

XML Treatment for Fritillaria maximowiczii

XML Treatment for Fritillaria usuriensis

XML Treatment for Lilium amabile

XML Treatment for Lycopodiaceae

XML Treatment for Huperzia asiatica

XML Treatment for Huperzia miyoshiana

XML Treatment for Huperzia selago

XML Treatment for Huperzia serrata

XML Treatment for Lythraceae

XML Treatment for Trapa incisa

XML Treatment for Malvaceae

XML Treatment for Tilia amurensis

XML Treatment for Nymphaeaceae

XML Treatment for Nelumbo nucifera

XML Treatment for Oleaceae

XML Treatment for Fraxinus mandshurica

XML Treatment for Orchidaceae

XML Treatment for Cypripedium calceolus

XML Treatment for Cypripedium guttatum

XML Treatment for Cypripedium macranthos

XML Treatment for Cypripedium shanxiense

XML Treatment for Cypripedium × ventricosum

XML Treatment for Gastrodia elata

XML Treatment for Gymnadenia conopsea

XML Treatment for Orobanchaceae

XML Treatment for Boschniakia rossica

XML Treatment for Pinaceae

XML Treatment for Pinus densiflora var. ussuriensis

XML Treatment for Pinus koraiensis

XML Treatment for Pinus sylvestris var. sylvestriformis

XML Treatment for Poaceae

XML Treatment for Coleanthus subtilis

XML Treatment for Zoysia sinica

XML Treatment for Rosaceae

XML Treatment for Malus komarovii

XML Treatment for Rosa rugosa

XML Treatment for Rutaceae

XML Treatment for Phellodendron amurense

XML Treatment for Scheuchzeriaceae

XML Treatment for Scheuchzeria palustris

XML Treatment for Sphagnaceae

XML Treatment for Sphagnum multifibrosum

XML Treatment for Sphagnum squarrosum

XML Treatment for Taxaceae

XML Treatment for Taxus cuspidata

XML Treatment for Tricholomataceae

XML Treatment for Tricholoma matsutake

XML Treatment for Typhaceae

XML Treatment for Sparganium hyperboreum

## Figures and Tables

**Figure 1.**
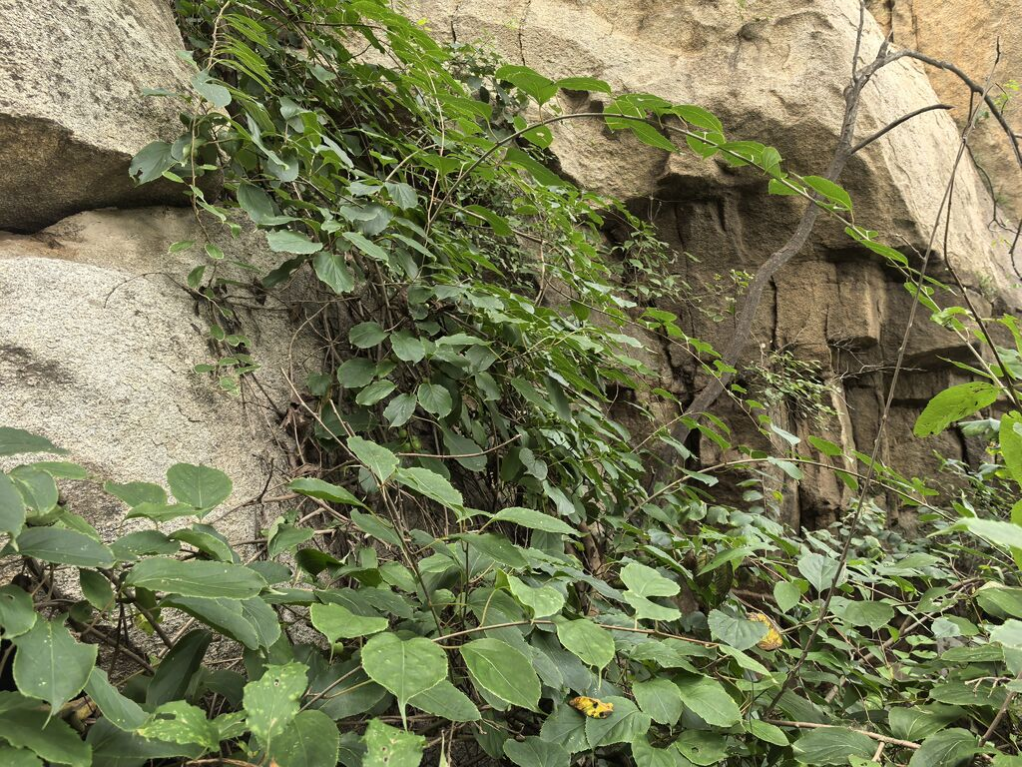
*Actinidiaarguta*.

**Figure 2.**
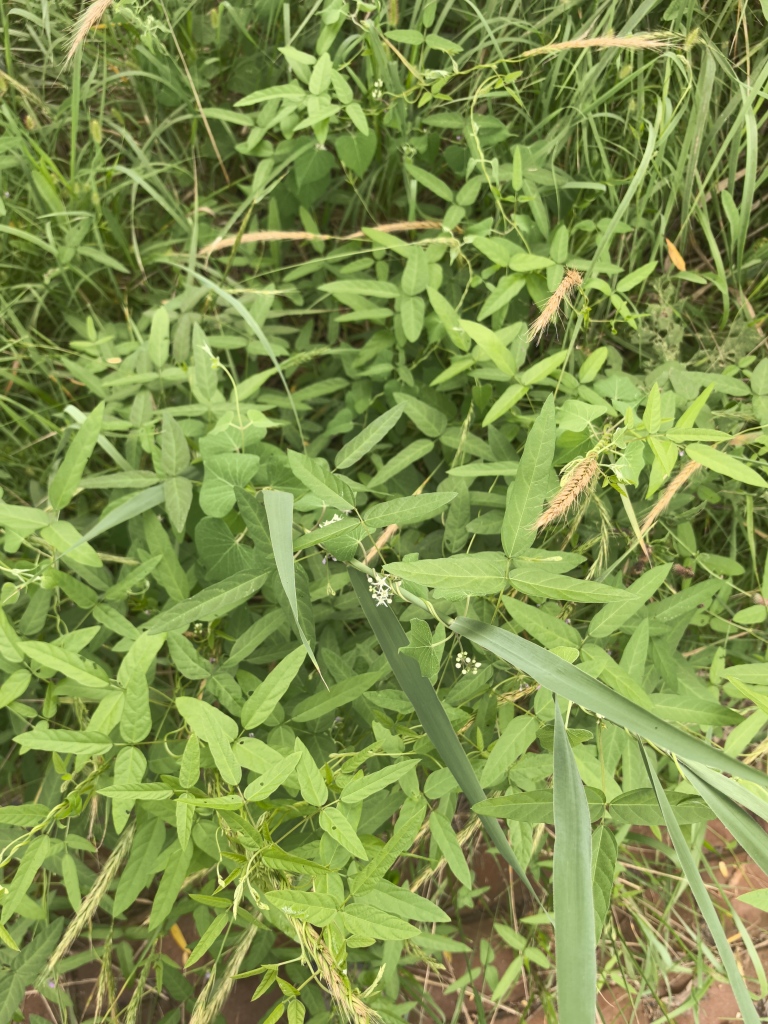
*Glycinesoja*.

**Figure 3.**
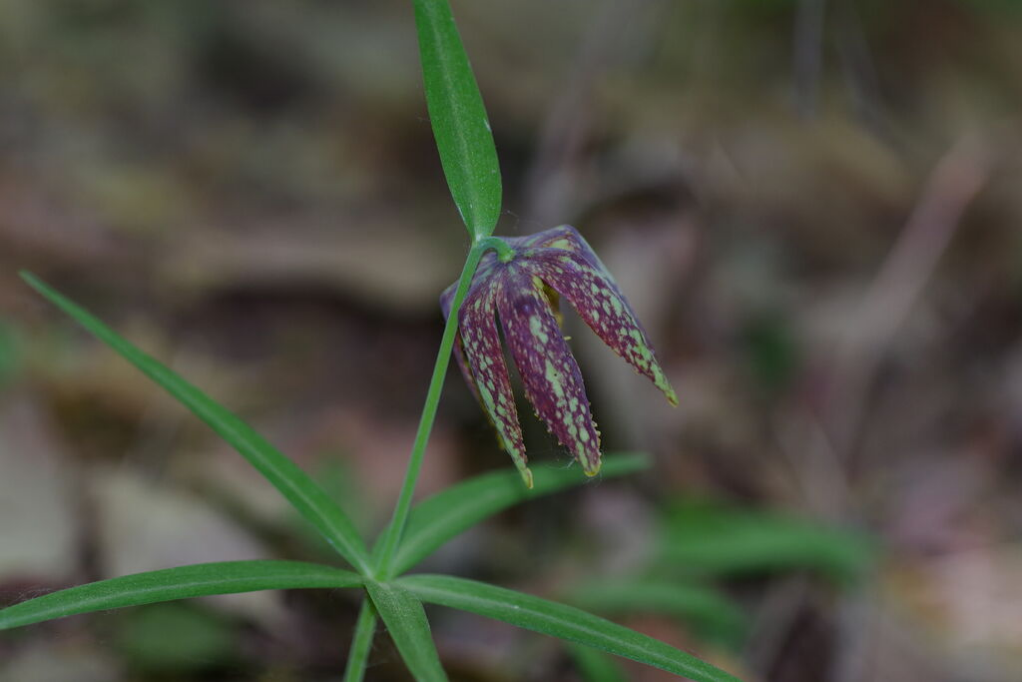
*Fritillariamaximowiczii*.

**Figure 4.**
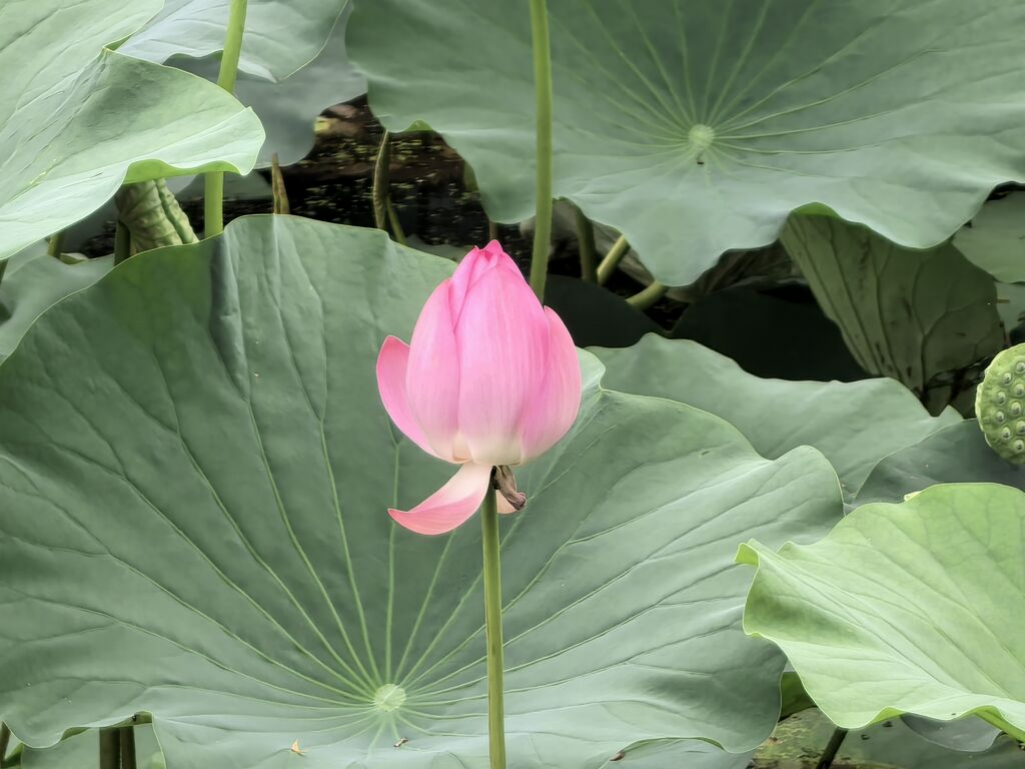
*Nelumbonucifera*.

**Figure 5.**
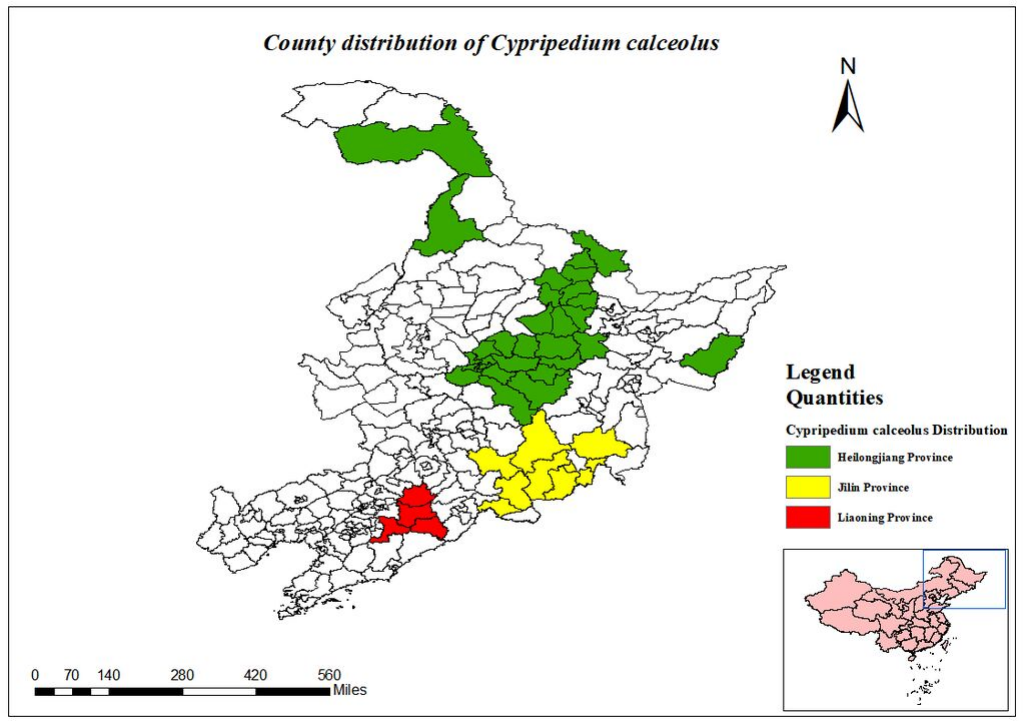
County distribution of *Cypripediumcalceolus*.

**Figure 6.**
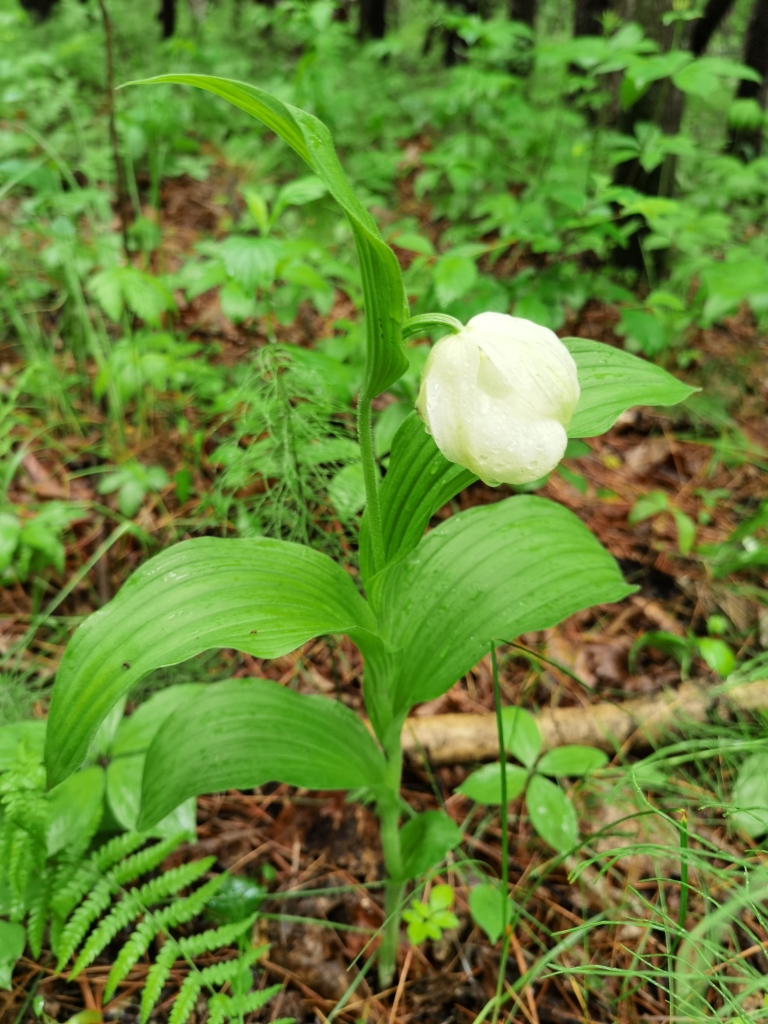
*Cypripediumcalceolus*.

**Figure 7.**
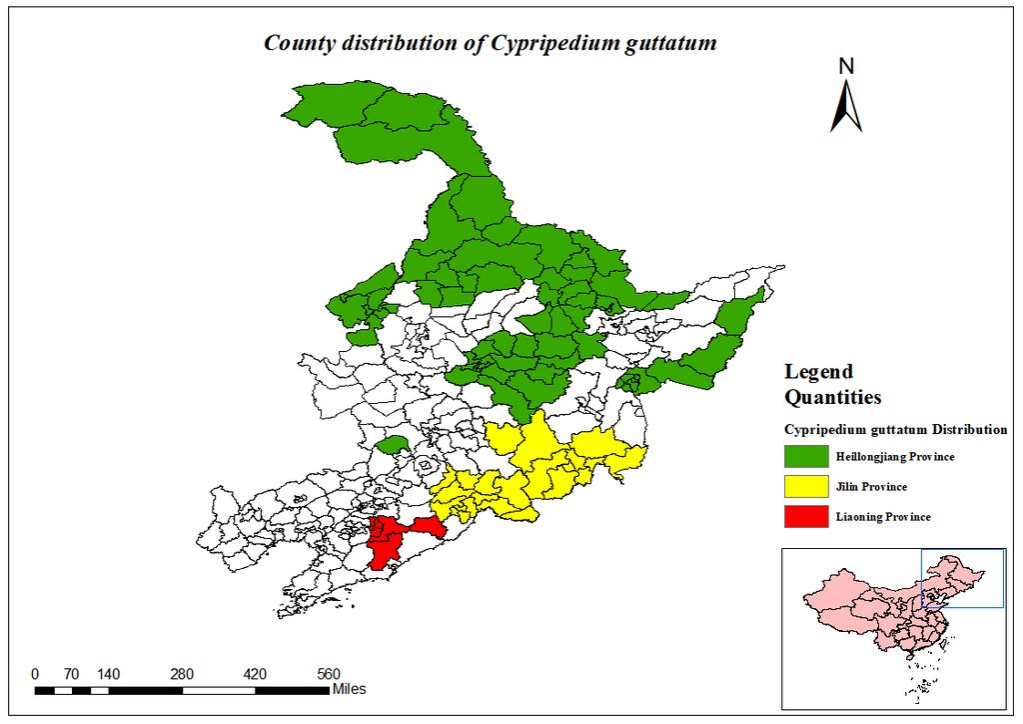
County distribution of *Cypripediumguttatum*.

**Figure 8.**
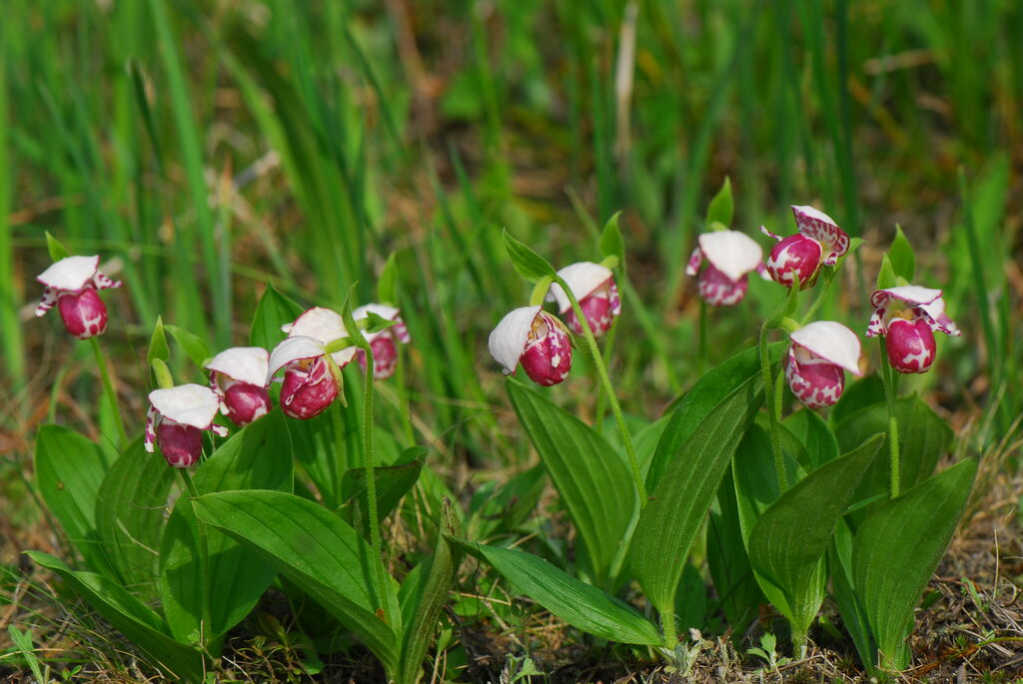
*Cypripediumguttatum*.

**Figure 9.**
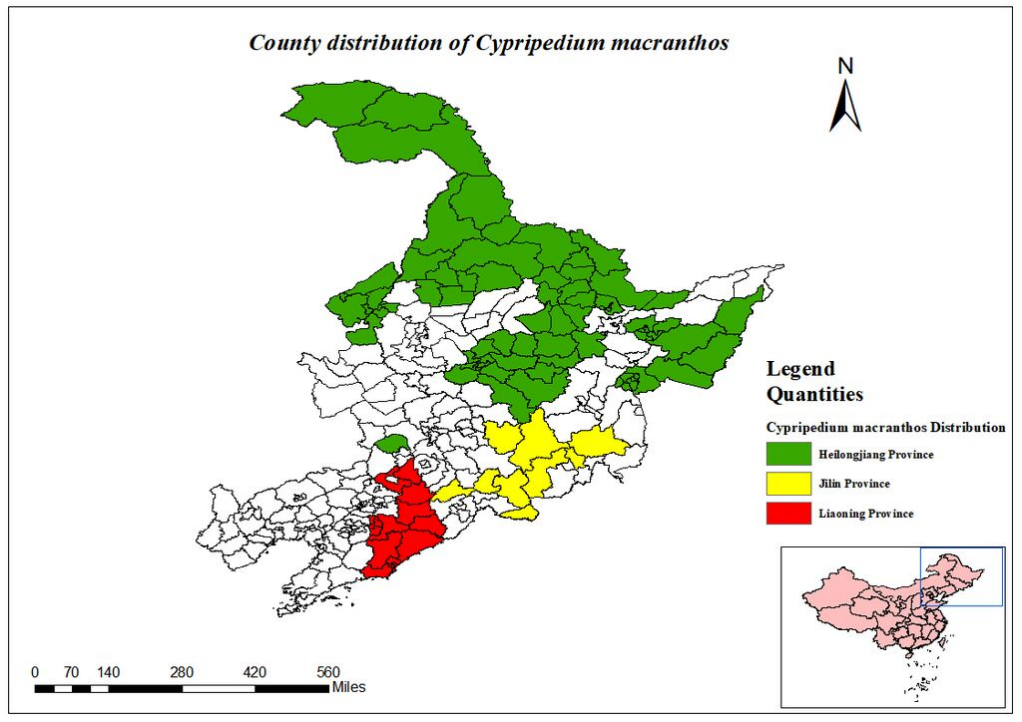
County distribution of *Cypripediummacranthos*.

**Figure 10.**
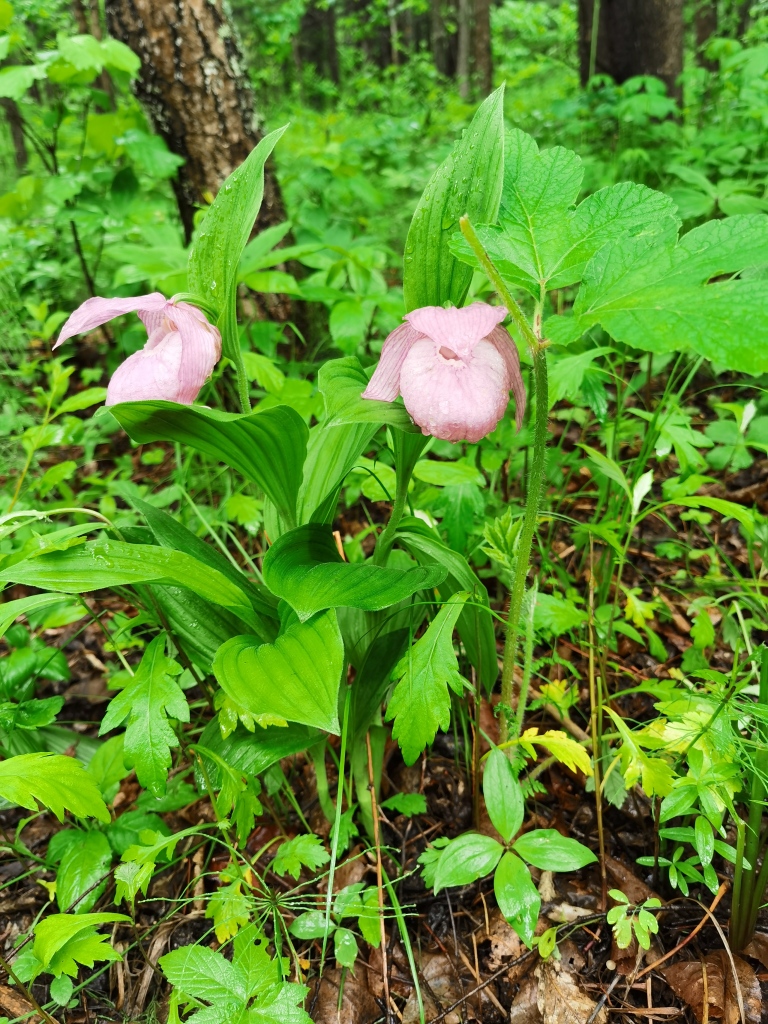
*Cypripediummacranthos*.

**Figure 11.**
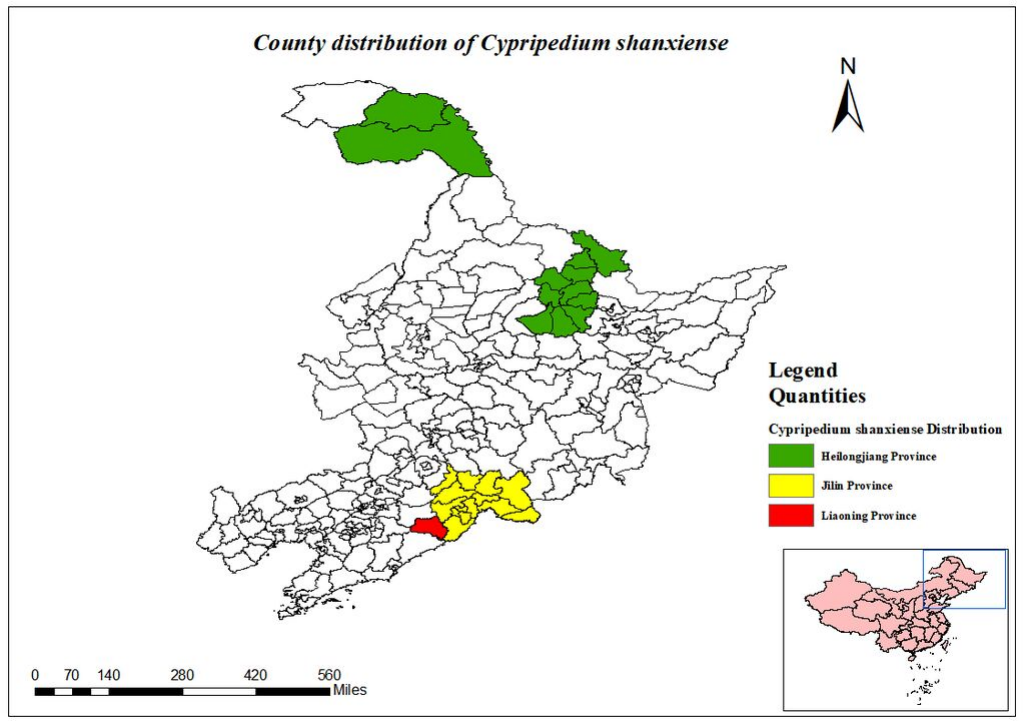
County distribution of *Cypripediumshanxiense*.

**Figure 12.**
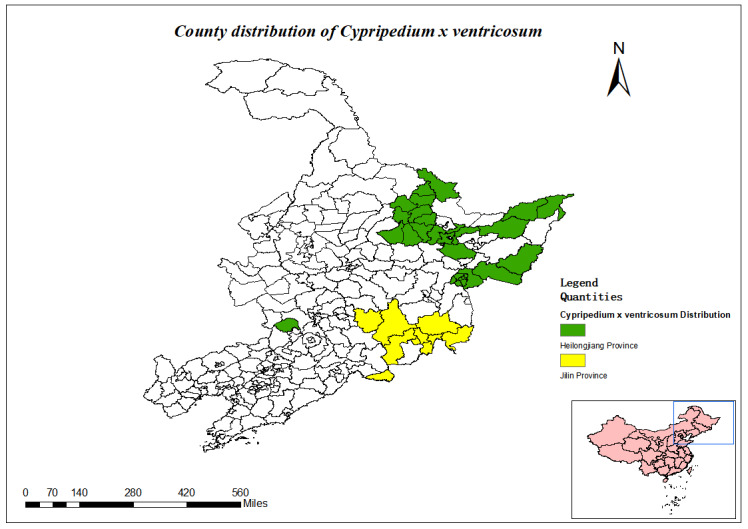
County distribution of *Cypripediumxventricosum*.

**Figure 13.**
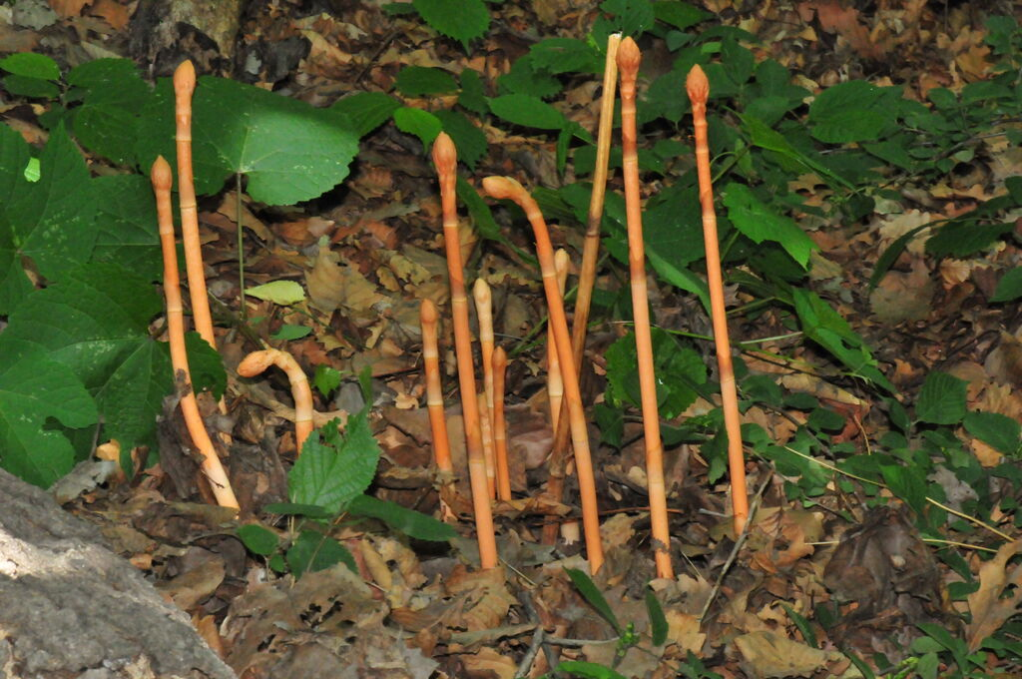
*Gastrodiaelata*.

**Figure 14.**
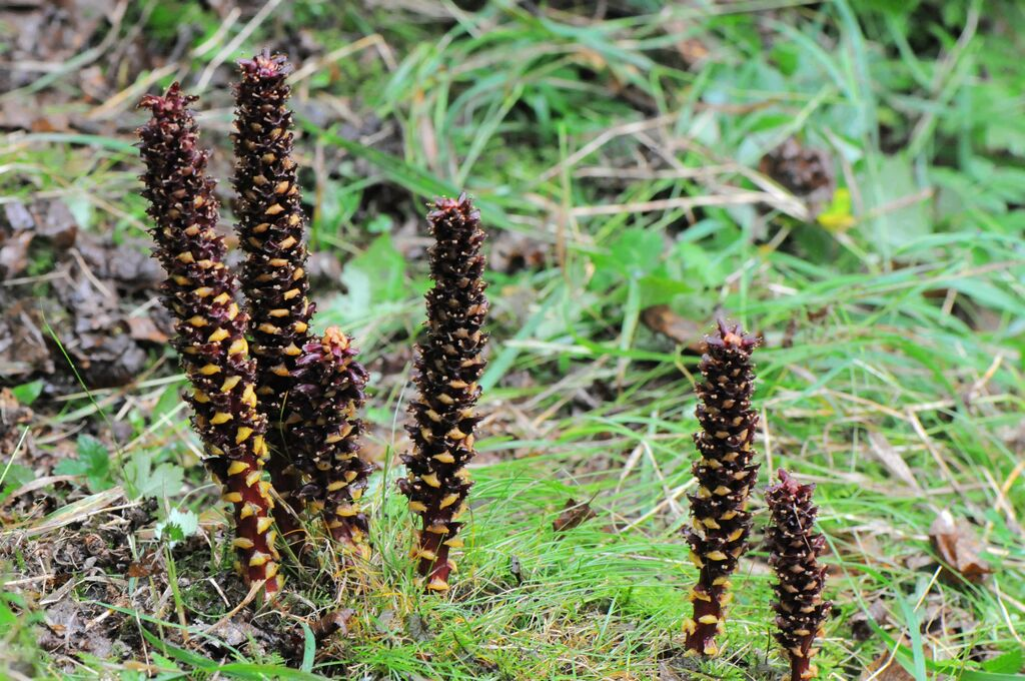
*Boschniakiarossica*.

**Figure 15.**
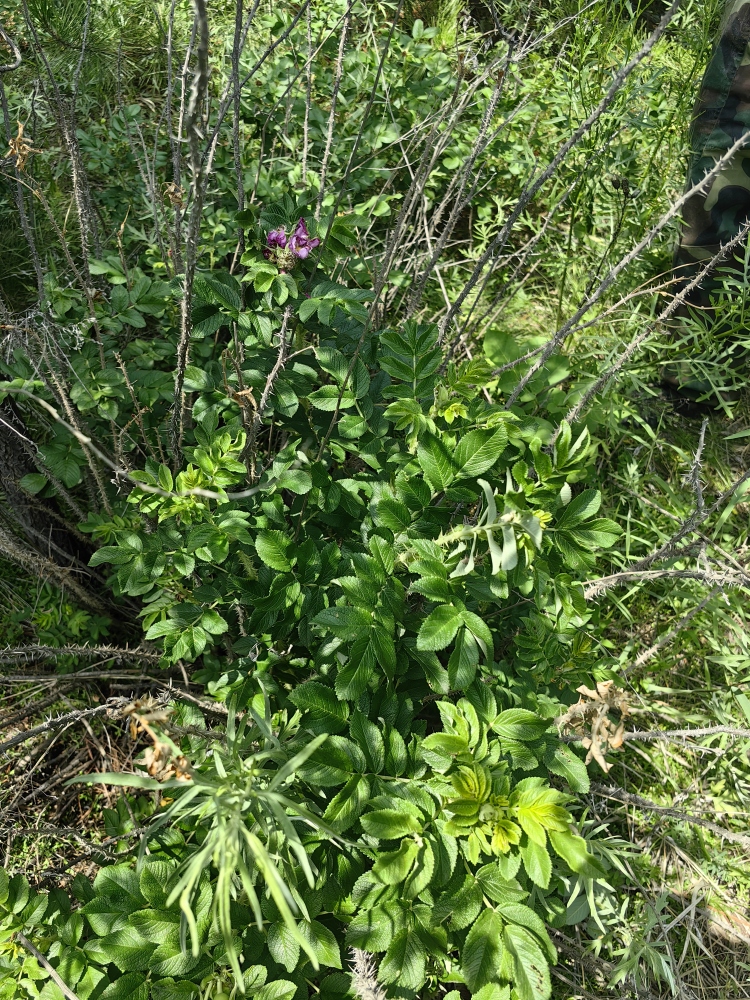
*Rosarugosa*.

**Figure 16.**
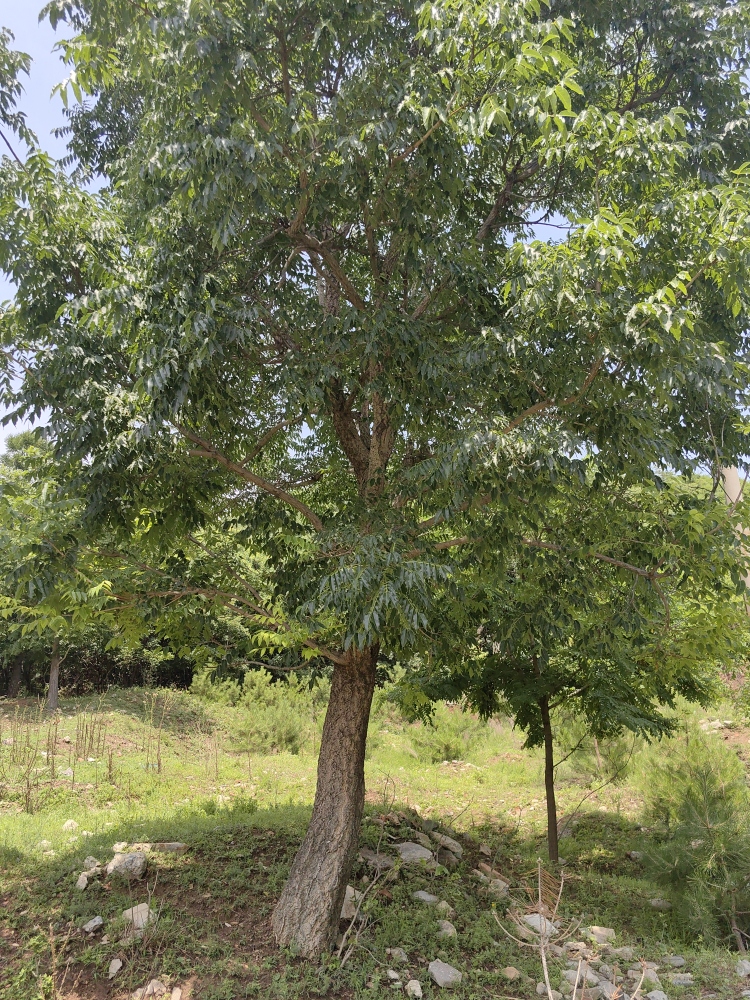
*Phellodendronamurense*.

**Figure 17.**
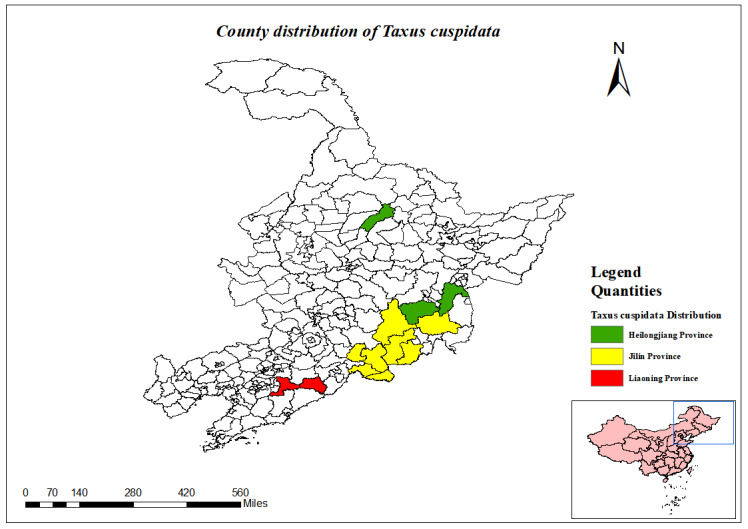
County distribution of *Taxuscuspidata*.

**Table 1.** Threat levels and threat factors of plants under state priority protection in the most north-eastern provinces of China.

Scientific name	Threat level	Threat factors
*Aldrovandavesiculosa*	Endangered	Residential & commercial development; Agriculture & aquaculture; Energy production & mining; Biological resource use; Natural system modifications; Pollution.
*Braseniaschreberi*	Least Concern	Uncharted
*Coleanthussubtilis*	Least Concern	Uncharted
*Cypripediumcalceolus*	Least Concern	Residential & commercial development; Agriculture & aquaculture; Biological resource use; Natural system modifications; Pollution.
*Cypripediumguttatum*	Least Concern	Residential & commericial development; Transportation & disturbance; Natural system modifications; Climate change & severe weather.
*Cypripediummacranthos*	Least Concern	Residential & commercial development; Agriculture & aquaculture; Transportation & service corridors; Biological resource use; Human intrusions & disturbance; Natural system modifications; Invasive and other problematic species.
*Cypripediumshanxiense*	Near Threatened	Residential & commercial development; Agriculture & aquaculture; Transportation & service corridors; Biological resource use; Human intrusions & disturbance; Pollution; Climate change & severe weather.
*Fraxinusmandshurica*	Least Concern	Residential & commercial development; Agriculture & aquaculture; Biological resource use; Climate change & severe weather.
*Gastrodiaelata*	Vulnerable	Uncharted
*Maluskomarovii*	Endangered	Residential & commercial development; Transportation & service corridors; Biological resource use; Human intrusions & disturbance; Natural system modifications; Pollution; Climate change & severe weather.
*Myriophyllumussuriense*	Least Concern	Uncharted
*Otteliaalismoides*	Least Concern	Biological resource use; Human intrustions & disturbance; Pollution.
*Pinuskoraiensis*	Least Concern	Biological resource use; Invasive and other problematic species.
*Scheuchzeriapalustris*	Least Concern	Uncharted
*Sparganiumhyperboreum*	Least Concern	Uncharted
*Taxuscuspidata*	Least Concern	Biological resource use.
*Thujakoraiensis*	Vulnerable	Biological resource use; Human intrusions & disturbance.
*Tiliaamurensis*	Least Concern	Residential & commercial development; Agriculture & aquaculture; Biological resource use.
*Trapaincisa*	Least Concern	Uncharted
